# Substituted adenine quartets: interplay between substituent effect, hydrogen bonding, and aromaticity†

**DOI:** 10.1039/d0ra04585c

**Published:** 2020-06-18

**Authors:** Halina Szatylowicz, Paulina H. Marek, Olga A. Stasyuk, Tadeusz M. Krygowski, Miquel Solà

**Affiliations:** Warsaw University of Technology, Faculty of Chemistry Noakowskiego 3 00-664 Warsaw Poland halina@ch.pw.edu.pl; University of Warsaw, Faculty of Chemistry Pasteura 1 02-093 Warsaw Poland; Department of Chemistry, Institute of Computational Chemistry and Catalysis, University of Girona C/ M. Aurèlia Capmany, 69 17003 Girona Spain o.a.stasuk@gmail.com

## Abstract

Adenine, one of the components of DNA/RNA helices, has the ability to form self-organizing structures with cyclic hydrogen bonds (A_4_), similar to guanine quartets. Here, we report a computational investigation of the effect of substituents (X = NO_2_, Cl, F, H, Me, and NH_2_) on the electronic structure of 9*H*-adenine and its quartets (A_4_-N1, A_4_-N3, and A_4_-N7). DFT calculations were used to show the relationships between the electronic nature of the substituents, strength of H-bonds in the quartets, and aromaticity of five- and six-membered rings of adenine. We demonstrated how the remote substituent X modifies the proton-donating properties of the NH_2_ group involved in the H-bonds within quartets and how the position of the substituent and its electronic nature affect the stability of the quartets. We also showed the possible changes in electronic properties of the substituent and aromaticity of adenine rings caused by tetramer formation. The results indicate that the observed relationships depend on the A_4_ type. Moreover, the same substituent can both strengthen and weaken intermolecular interactions, depending on the substitution position.

## Abstract 2

Substituent effects on hydrogen bonds in adenine quartets and aromaticity of adenine rings depend on the quartet type. A_4_-N3 and A_4_-N7 quartets are more responsive to the electronic nature of substituents than A_4_-N1.

## Graphical abstract

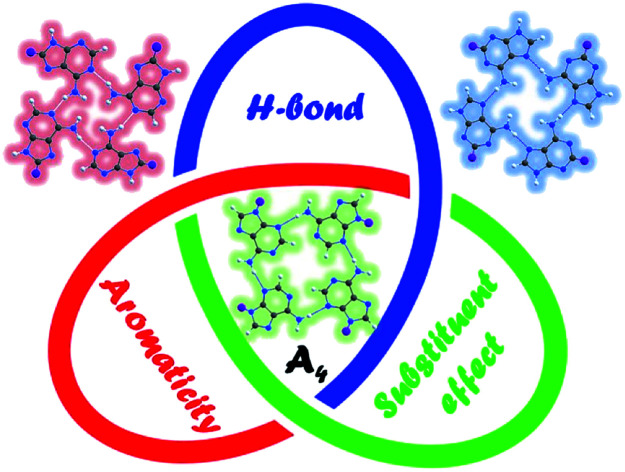

## Introduction

Purine derivatives play a significant role in medicinal chemistry and they are also important intermediates for the synthesis of biologically-active nucleoside analogues, and frequently exhibit antiviral or antitumor properties.^1^ The nature and position of the substituents determine their reactivity and biological activity. Adenine (6-amino substituted purine derivative), one of the building blocks of DNA/RNA helices, can participate in intermolecular interactions which may lead to substantial changes in its electronic structure and, in consequence, to changes in its chemical/physicochemical/biochemical properties. In this perspective, the knowledge of the changes in the electronic structure of adenine caused by well-defined factors, such as introduction of substituents with specific electron donating/accepting properties, is of fundamental importance. It should be stressed that the effects of the intermolecular interactions (H-bonding, π-stacking, *etc.*) on the electronic structure and molecular properties of nucleic acid bases have long been a subject of wide and intensive studies.^2^ However, the effects of substituents on the properties of nucleic acid bases are much less represented in the literature. It was reported that in the synthesis of deoxyribonucleosides, substituents at the C8 position of purine can greatly affect the rate of deoxyribosyl transfer to the base and the nature of the nucleoside formation.^3^ An electron-donating substituent directs deoxyribosyl transfer to N9 of the base, while an electron-withdrawing substituent leads to the N3-product. Halogen substituents at the C8 position of adenine do not change the tautomeric preference in the gas phase but induce significant changes in vibrational frequencies and Raman intensities.^4^ Substituent effects on hydrogen bonding were most frequently studied in structurally-modified Watson–Crick base pairs (adenine–thymine and guanine–cytosine).^5–11^

One of the interesting features of DNA/RNA bases is their ability to form self-assembled structures (clusters). Among them, the most commonly studied are cyclic hydrogen-bonded quartets (tetramers),^12,13^ which occur naturally in telomeres,^14^ immunoglobulin gene switch regions,^15^ and certain gene promoters.^16^ The guanine quartet is the most well-known for a long time, for reviews see ref. 17 and 18. Although adenine quartets (A_4_) have been studied experimentally^19–22^ and have also been the subject of computational investigations,^23–26^ the effect of remote substituents on hydrogen bonds in adenine tetramers has not yet been studied. Introduction of additional functionalities to the adenine with subsequent formation of quartet with intermolecular adenine⋯adenine hydrogen-bond interactions is one of the design strategies to enrich chemistry of purine derivatives with ability to bind many metal ions.^27^ Since the stabilization of such complexes depends on the functional groups, the simulation of adenine quartets with certain substituents of different electronic nature is a good step toward design of synthetic functional materials. Moreover, changes in aromaticity of the adenine rings in the tetramer could be used to fine-tune π–π stacking interactions.

In this work, we have investigated computationally the effect of substituents (X = NO_2_, Cl, F, H, Me, NH_2_) on electronic structure of 9*H*-adenine and its quartets (A_4_-N1, A_4_-N3, and A_4_-N7) with dispersion-corrected Density Functional Theory. We have demonstrated how the position of the substituent and its electronic nature affect the stability of the quartets. Additionally, we have analyzed the influence of the electronic nature of the substituents on the strength of H-bonds in the quartets and aromaticity of five- and six-membered rings of adenine.

## Methodology

**Fig. 1:**
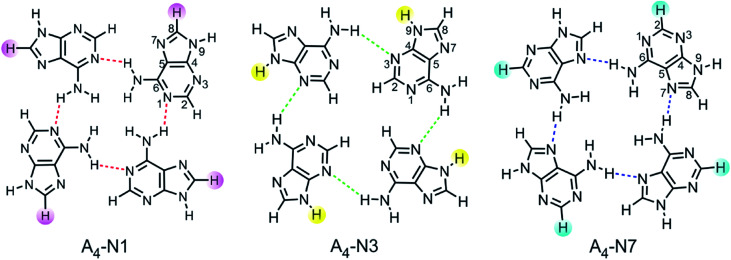
Analyzed tetramers of adenine with places of substitution marked, C8, N9, and C2, respectively.

Quartets of substituted 9*H* adenine tautomer were chosen as models for studying interrelationships between electronic nature of substituents, aromaticity of transmitting moiety, and intermolecular H-bonding between individual adenine molecules. Three types of adenine quartets were considered: A_4_-N1, A_4_-N3 and A_4_-N7. They are formed by one hydrogen bond between two neighbouring bases as shown in Fig. 1. These quartets have a non-planar *S*_4_-symmetric structure as the global minimum. However, they are forced into planarity by stacking interactions which compensate for the energy spent on planarization. The planarization energy of A_4_-N3 and A_4_-N7 was found to be 0.2 and 11 kcal mol^−1^, respectively, while for A_4_-N1 system it was 22.3 kcal mol^−1^.^28^ The low stability of planar A_4_-N1 quartet is probably a main reason why this type of quartet has not been characterized by X-ray crystallography in contrast to A_4_-N3 ^29^ and A_4_-N7.^20^

Selected substituents, varying in their electronic properties (X = NO_2_, Cl, F, H, Me, NH_2_), were inserted into each adenine molecule at the same position within a certain quartet. For A_4_-N1 and A_4_-N3 tetramers, it was done either at the C8 or N9 position, whereas in the case of A_4_-N7 tetramer they were introduced either at the C2 or N9 position, resulting with 33 systems to be analysed. Although N9 substitution is less common, some organic ligands with functional groups can be incorporated into the adenine molecule at the N9 position.^27^

Geometry optimization of each system was performed using ADF program^30–32^ at the BLYP/TZ2P level of theory^33–35^ with Grimme D3 dispersion correction.^36,37^ To ensure that the obtained geometries are in energy minima, the vibrational analysis was performed and no imaginary frequencies were found.

The bonding energy of the tetramers, or hydrogen bond energy, *E*_HB_, was calculated as sum of interaction and preparation energies.

1 *E*_HB_ = *E*_int_ + *E*_prep_

Interaction energy, *E*_int_, was calculated as the difference between the energy of the tetramer and the sum of the energies of its components (adenines) in the tetramer geometry.

2 *E*_int_ = *E*_tetramer_ − 4*E*_monomer in tetramer geometry_

Preparation energy, *E*_prep_, was calculated as the difference between energies of adenine monomers in the tetramer geometry and adenine monomers in equilibrium geometry.

3 *E*_prep_ = 4*E*_monomer in tetramer geometry_ − 4*E*_monomer_

Analysis of intermolecular interactions within the framework of Bader's Quantum Theory of Atoms in Molecules (QTAIM) was performed using the AIMAll program package.^38^ Such parameters as the electron density in bond critical points (BCPs), *ρ*_BCP_, its Laplacian, ∇^2^*ρ*_BCP_, density of the total electron energy in BCP, *H*_BCP_, and its two components, potential and kinetic electron energy densities, *V*_BCP_ and *G*_BCP_, respectively, were taken into account.

Substituent effect was described using cSAR (charge of the Substituent Active Region) concept.^39,40^ The cSAR(X) value was calculated as the sum of Voronoi charges^41^ of the atoms of substituent X and *ipso* C- or N-atom of adenine.

4 cSAR(X) = *q*(X) + *q*(C_*ipso*_ or N_*ipso*_)

The choice of VDD charges is based on our previous research into the electronic structure of the nitro group in *para*-substituted nitrobenzene derivatives^42^ and studies of adenine systems substituted in C-*X* and N-*X* positions.^43^

Electron delocalization was characterized using geometry-based aromaticity index HOMA (Harmonic Oscillator Model of Aromaticity),^44,45^ which is defined as:5
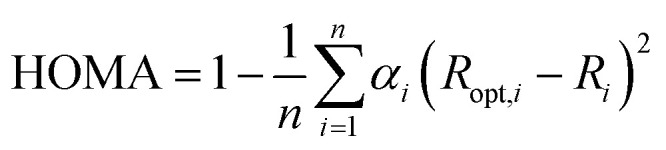
where *n* is the number of bonds taken into the summation; *α*_*i*_ is a normalization constant (for CC and CN bonds, *α*_CC_ = 257.7 and *α*_CN_ = 93.52) fixed to give HOMA = 0 for a model non-aromatic system and HOMA = 1 for the system, in which all bonds are equal to the optimal value *R*_opt,*i*_ assumed to be realized for fully aromatic systems (for CC and CN bonds *R*_opt,CC_ = 1.388 Å and *R*_opt,CN_ = 1.334 Å) and *R*_*i*_ denotes bond lengths taken into calculation.

The local HOMA index was calculated for both five- and six-membered adenine rings. This aromaticity descriptor was previously successfully used to describe the aromaticity changes in adenine rings due to H-bonding and complexation of metal ions.^46^ The obtained values of the bonding energies and all substituent effect descriptors are gathered in Tables S1–S4.† cSAR and HOMA parameters were calculated for each individual adenine molecule in the tetramer. Because their values are similar within a given tetramer for all molecules, the mean values are given in the Tables S2 and S4,† respectively.

## Results and discussion

The optimized A_4_-N3 and A_4_-N7 structures with substituents resemble flat geometry in symmetry close to *C*_4h_, while the substituted A_4_-N1 quartets are more distorted and adopt a similar pattern to that described by the *S*_4_ point group. The unconstrained optimized geometries of systems with different substituents do not change significantly within one quartet type (Fig. S1†). The obtained H-bond lengths for unsubstituted A_4_-N3 (*d*_N⋯N_ = 3.06 Å) and A_4_-N7 (*d*_N⋯N_ = 2.93 Å) are close to the experimental values, 3.05 and 2.91 Å for A_4_-N3 and A_4_-N7, respectively.^20,29^ The three quartets have similar stabilities. Our calculations show that unsubstituted A_4_-N3 tetramer is the most stable followed by A_4_-N7 at only 0.4 kcal mol^−1^ and A_4_-N1 at 2.7 kcal mol^−1^ (Table S1†). However, substituents affect the electronic structure of the tetramers and change this stability order. In general, among N9-substituted tetramers the A_4_-N3 is the most stable quartet. For C2/C8-substituted quartets, the A_4_-N3 tetramer is more favourable only with H and Me substituents, while for the other studied substituents the A_4_-N7 tetramer is preferred. It should be noted that the most stable substituted A_4_-N7 tetramers (except X = H and Me) have the shortest hydrogen bonds (*d*_N⋯H_ < 1.9 Å). According to the substitution position, C2/C8-substituted tetramers are considerably more stable than the N9-substituted ones.

Intramolecular interactions, the so-called substituent effect, can be considered from three points of view, as classical and reverse substituent effects, as well as the influence of the substituent on the properties of transmitting moiety. The classical substituent effect, introduced and quantified by Hammett,^47,48^ characterizes how a substituent X affects properties of a fixed group Y (so-called “reaction site”) in a substituted system X–R–Y (R – transmitting moiety). In our case, properties of the reaction site – the amino group – can be described either by the cSAR(NH_2_) value or by the strength of H-bonds.

### Substituent effect on H-bonding – classical substituent effect

**Fig. 2:**
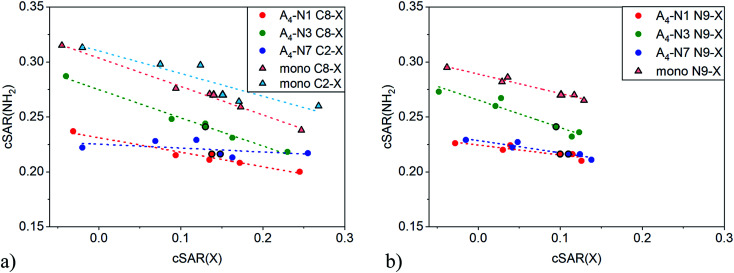
Dependences of cSAR(NH_2_) on cSAR(X) for C2-/C8- (a) and N9- (b) substituted adenine monomers and tetramers. More positive cSAR(X) values correspond to more electron-donating substituents.

An illustrative example of the classical substituent effect is the dependence of cSAR(NH_2_) on cSAR(X) shown in Fig. 2. The dependence was considered not only for quartets but also for substituted adenine monomers. Slopes of linear equations and determination coefficients are presented in Table 1. Note that correlations are acceptable or even good with *R*^2^ > 0.84, except for C2-substituted A_4_-N7 series, where very narrow range of cSAR(NH_2_) values was found. In all cases the slope *a* is negative meaning that electron-donating properties of the amino group decrease with increasing electron-donating ability (more positive cSAR(X) value) of substituent X. According to the slope *a* value, the C8 position in monomer is the most sensitive to the electronic nature of substituent X. This is consistent with results for adenine based on other substituent effect descriptors.^49^

**Table 1:** Slope values, *a*, and determination coefficients, *R*^2^, of cSAR(NH_2_) *vs.* cSAR(X) linear regressions for substituted adenine monomers and tetramers shown in Fig. 2

Position	C2/C8		N9	
	*a*	*R* ^2^	*a*	*R* ^2^
Monomer C2	−0.206	0.835	—	—
Monomer C8	−0.259	0.992	—	—
Monomer N9	—	—	−0.176	0.969
A_4_-N1	−0.133	0.957	−0.090	0.845
A_4_-N3	−0.257	0.987	−0.250	0.921
A_4_-N7	−0.036	0.248	−0.112	0.896

As expected, cSAR(NH_2_) values are higher in monomers (triangles) than in quartets (circles) where amino group is involved in H-bond. The reason is accumulation of negative charge on the N atom of proton-donating –NH part of the amino group during formation of hydrogen bond. As can be seen in Fig. 2, electron-donating power of the NH_2_ group is less affected by the tetramer formation in A_4_-N3 quartets than in the others, because the formed H-bonds in A_4_-N3 are the longest among all quartets, *d*_N⋯H_ > 2.0 Å. This observation was also confirmed by direct comparison of cSAR(NH_2_) of NH_2_ attached to C6 in monomers and tetramers, see Fig. S2 and Table S5.† In A_4_-N3, cSAR(NH_2_) values of monomers and tetramers are comparable, while for A_4_-N1 and A_4_-N7, cSAR(NH_2_) in tetramers are significantly smaller than in monomers. Moreover, A_4_-N3 quartet is the most responsive to the electronic nature of substituent X either at C8 or N9 position.

**Fig. 3:**
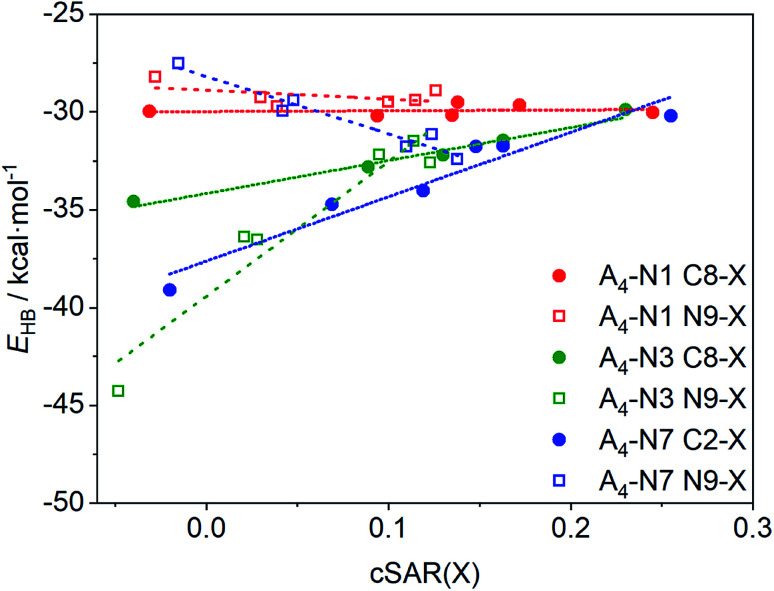
Relationships between the bonding energies, *E*_HB_, and cSAR(X) of substituent for the studied adenine quartets.

As a consequence of the substituent effect on the properties of the amino group, H-bond energy in quartets also changes. Fig. 3 shows the dependence of bonding energy, *E*_HB_, in A_4_-N1, A_4_-N3, and A_4_-N7 tetramers on the electronic properties of remote substituents, where substituent X is described by cSAR(X) value. More positive cSAR values indicate better electron-donating properties of substituents. According to Fig. 2, substituent X changes properties of the amino group in the same direction regardless to the quartet type. However, the substituent effect on H-bond energy is different. This means that in addition to the substituent effect on the amino group, the substituent X also affects the proton-accepting abilities of N atoms of both rings. Positive and negative slopes of linear equations (Table 2) show the direction of the substituent effect, whereas their magnitude displays a sensitivity of intermolecular interactions to electronic properties of the substituents X.

**Table 2:** Slope values, *a*, and determination coefficients, *R*^2^, of the linear regressions *E*_HB_*vs.* cSAR(X), shown in Fig. 3

Substitution position	C2/C8		N9	
Quartet type	*a*	*R* ^2^	*a*	*R* ^2^
A_4_-N1	0.413	0.018	−4.468	0.244
A_4_-N3	16.86	0.970	68.91	0.921
A_4_-N7	32.91	0.934	−29.23	0.936

Two groups can be distinguished in the studied systems: (i) with positive slope, where H-bond weakens with increase in electron-donating properties of substituent X, and (ii) with negative slope, where H-bond strengthens with the same substituent change. The first group consists of all adenine quartets with the substituents at the carbon atom (either C2 or C8) and N9-substituted A_4_-N3 quartet. The second group is represented by A_4_-N1 and A_4_-N7 quartets with the N9-substituents. In the case of A_4_-N3 and A_4_-N7 tetramers (*R*^2^ > 0.92), the substituents notably change the energy of the intermolecular interactions. The largest changes were found in the N9-substituted A_4_-N3 system. This is consistent with observation of substituent effect X on properties of the NH_2_ group (Fig. 2). On the other hand, for substituted A_4_-N1 quartets the changes in the H-bonds strength caused by the substituent X at any position are very small (slopes *a* are only 0.413 and −4.468, respectively) and *R*^2^ of these regressions are less than 0.25. Such low coefficients of determination (Table 2) can be explained by very narrow energy range. This is due to the fact that both C8 and N9 substitution positions in A_4_-N1 quartets are far from the atoms participated in the H-bond formation and the substituent effect is not transmitted so far. Different slopes for the A_4_-N7 substituted tetramers can be explained as follows: when substituent X is attached to the N9 position it changes the electron density on the N7 atom and modifies its proton-accepting power to a greater extent, increasing *E*_HB_ for electron-donating X groups, whereas the substitution at the C2 position affects mostly the proton-donating ability of the amino group decreasing *E*_HB_ for electron-donating X groups. Thus, depending on the place of substitution the same substituent X can either weaken or enhance the intermolecular H-bonds in A_4_-N7 tetramers.

Summarizing, the strong electron-accepting substituents contribute to strengthening of H-bonds in A_4_-N3 (at the C8 and N9 position) and A_4_-N7 (at the C2 position) quartets. The electron-donating substituents make H-bonds stronger only in A_4_-N7 quartet if they are located at the N9 position. Introducing different substituents into A_4_-N1 quartet has relatively small effect.

### Additional intermolecular interactions

**Fig. 4:**
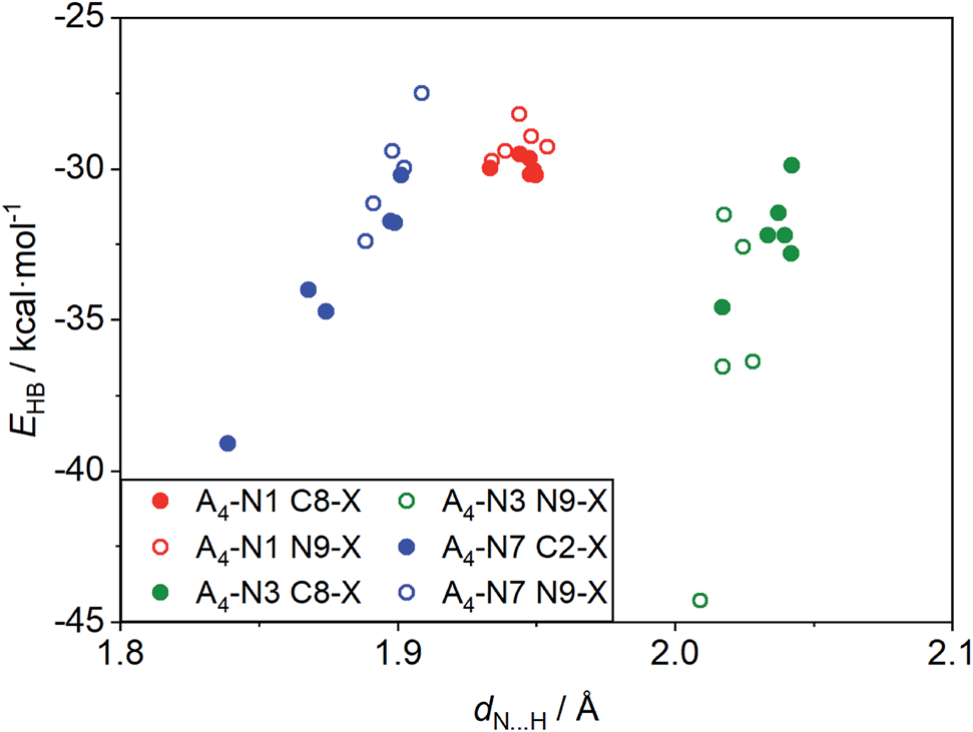
Relationships between bonding energy and N⋯H length, *d*_N⋯H_, for the studied substituted tetramers.

**Fig. 5:**
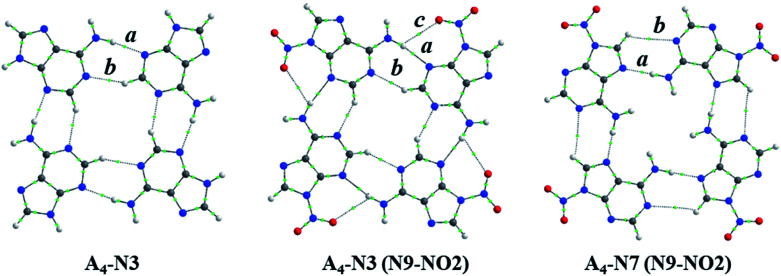
Structures of A_4_-N3, A_4_-N3(N9-NO_2_) and A_4_-N7(N9-NO_2_) systems with bond critical points: *a* is a typical H-bond in quartet, *b* – additional CH⋯N contact, *c* – additional O⋯HN contact.

Some interesting observation was done when considering the relationships between strength of intermolecular interactions and length of the hydrogen bonds, *d*_N⋯H_, shown in Fig. 4. Despite the fact that H-bond lengths in A_4_-N3 quartets are longer (*d*_N⋯H_ > 2.0 Å) than in the A_4_-N7 ones (*d*_N⋯H_ < 1.9 Å), the bonding energies in A_4_-N3 tetramers are comparable in most cases with the energies in A_4_-N7 systems or even stronger. It is worth mentioning that the two systems with the strongest and the weakest H-bonds have a common element, namely the nitro group as a substituent. These terminal cases were found in the A_4_-N3 and A_4_-N7 tetramers with the nitro substituent at the N9 position (Fig. 5). Their energies are −44.3 and −27.5 kcal mol^−1^ for A_4_-N3 and A_4_-N7, although H-bond lengths are 2.01 and 1.91 Å, respectively. The reduced *E*_HB_ in A_4_-N7 N9-NO_2_ substituted tetramer is expected because of the electron-accepting character of the nitro group as discussed above, whereas the large *E*_HB_ in A_4_-N3 N9-NO_2_ substituted tetramer is understood not only by the electron-accepting character of the substituent (*vide supra*), but also by the presence of additional intermolecular interactions.

Upon a detailed examination of their structures we found two additional close contacts in A_4_-N3 quartets (Fig. 5). One of them is formed between C–H unit of one adenine molecule and N1 atom of the other adenine (CH⋯N), whereas the second contact is made between NO_2_ group and NH_2_ group (O⋯HN). To characterize these extra contacts quantitatively we performed QTAIM analysis for 3 systems: unsubstituted A_4_-N3, and N9-NO_2_-substituted A_4_-N3 and A_4_-N7. Particular attention was paid to CH⋯N interactions in A_4_-N3 quartets. The distance *d*_H⋯N_ is 2.33 Å in A_4_-N3, and 2.27 Å in its N9-NO_2_-substituted analogue, whereas such *d*_H⋯N_ in adenine–thymine pair is 2.75 Å. The parameters of the corresponding BCPs are collected in Table 3. Their values confirm that CH⋯N and O⋯HN contacts are weak closed-shell interactions. Although the interactions between NO_2_ and NH_2_ groups are very week, the CH⋯N contacts in A_4_-N3 quartets can be considered as weak H-bonds,^50^ and therefore additionally contribute to their stability.

**Table 3:** QTAIM characteristics for noncovalent interactions in A_4_-N3, A_4_-N3(N9-NO_2_), and A_4_-N7(N9-NO_2_) systems

BCP	Atoms	*d*_H⋯N_ Å^−1^	*ρ*_BCP_	∇^2^*ρ*_BCP_	*V*	*G*	*H*	|*V*|/*G*	DI
**A**_**4**_ **-N3(N9-H)**									
a	N–H	2.03	0.024	0.077	−0.016	0.018	0.001	0.921	0.080
b	N–H	2.33	0.012	0.056	−0.008	0.011	0.003	0.754	0.041

**A**_**4**_ **-N3(N9-NO**_**2**_ **)**									
a	N–H	2.01	0.025	0.079	−0.017	0.019	0.001	0.936	0.082
b	N–H	2.27	0.014	0.063	−0.010	0.013	0.003	0.782	0.046
c	O–H	2.67	0.003	0.024	−0.002	0.004	0.002	0.565	0.004

**A**_**4**_ **-N7(N9-NO**_**2**_ **)**									
a	N–H	1.91	0.033	0.095	−0.023	0.023	0.000	0.989	0.104
b	N–H	3.07	0.002	0.011	−0.001	0.002	0.001	0.559	0.005

### Change in substituent electronic properties – reverse substituent effect

The cSAR concept allows us to study the reverse substituent effect,^42^ which describes how the electronic properties of substituents X depend on the properties of the moiety R–Y to which they are attached. Various substituent constant scales^51–53^ “represent” a fixed electronic structure of substituents or, in other words, its fixed electron-donating/attracting power. In turn, cSAR(X) value characterizes not the fixed electronic properties of substituent X but shows its properties depending on the substituted moiety. The reverse substituent effect can be illustrated by changes in cSAR(X) values for a particular substituent X when it is considered in different adenine quartets (Fig. S3†). The range of cSAR(X) values (*Δ* in Table S4†) documents that the properties of X depend on the moiety to which it is attached. For C-substituted systems, the largest change is observed for the NH_2_ substituent, while for the N-substituted ones the cSAR(NO_2_) changes the most; these changes correspond to approximately 13 and 20% of the total variation of the cSAR(X) values in the series, respectively.

The effect of tetramer formation on the electronic properties of X can be demonstrated by comparing cSAR(X) in the quartet with the values in the adenine monomer (Fig. S4 and Table S6†). Undoubtedly, very high determination coefficients, *R*^2^ > 0.99 and slopes close to 1.0 indicate a large similarity of interactions between X and substituted moieties in both cases. For N9-substitution in A_4_-N3 systems, the slope *a* is slightly larger than in other series. This means that in this case the substituent X is more responsive to tetramer formation most probably due to additional spatial interactions with the amino group of neighboring adenine molecule (Fig. 5). In adenine monomer, cSAR(X) values of all substituents are slightly greater than their values in tetramers (Table S4†), where these values depend on the position of substituent X relative to the proton-acceptor place (N1, N3 or N7) and proton-donor group (NH_2_). Only in N9-substituted A_4_-N7 tetramer, cSAR(X) values become more positive than in monomer due to stronger withdrawal of electronic density to the H-bonding area. Importantly, the H-bond formation may decrease the electron-attracting ability of the nitro group by ∼60%.

### Substituent effect on aromaticity

The substituent effect on aromaticity of the transmitting moiety can be demonstrated by changes in HOMA index for five- and six-membered rings in the substituted quartets, as presented in Fig. S5 and Table S7.† Correlations between HOMA indices and cSAR(X) values indicate that electron-donating substituents at the C2 and C8 positions decrease the aromaticity of the five-membered rings, while at the N9 position cause the opposite effect; the latter is in line with previous studies^54^ showing that N-substitution in imidazole rings increases the aromaticity for electron-donating groups. The six-membered rings are highly aromatic (HOMA > 0.9) and is slightly responsive only to the substituents at the C8 position, whereas C2- and N9-substitution does not significantly affect them. Increase in the aromaticity of the five-membered ring by substitution, in general, goes with a reduction of the aromaticity of the six-membered ring. Aromaticity changes by N9-substitution in the five-membered ring translate into small variation in the six-membered ring. Moreover, minor aromaticity changes by C2-substitution are in line with previous studies indicating a high resistance of the π-electron structure to the substituent effect.^55^ According to the slopes *a*, the highest HOMA sensitivity to the substituent effect was observed in A_4_-N3 quartets, most likely due to multiple noncovalent interactions between adenine molecules. Different slopes observed for five- and six-membered rings may depend on the number of bonds between the substituent X and the “reaction site” (NH_2_). A similar observation is well known for *meta* and *para* substituted benzene derivatives, where the substituent effect depends on the number of bonds between X and Y.^56^

Comparison of HOMA values for tetramers with values for monomers of substituted adenine (Table S3†) clearly shows that aromaticity of both rings slightly decreases upon tetramer formation. The five-membered rings of A_4_-N7 quartet become more aromatic than in monomer because their N7 atom participates in the H-bond as a proton-acceptor. Great correlations between HOMA values of five-membered rings for substituted monomers and tetramers (*R*^2^ > 0.94; Fig. S6 and Table S8†) with slopes *a* close to 1.0 point out that aromaticity of five-membered rings does not change much upon tetramer formation. Aromaticity of six-membered rings in tetramer becomes either more sensitive (*a* > 1.0) or less sensitive (*a* < 1.0) to the nature of substituent X in comparison with monomer.

## Conclusions

The substituent X, depending on the position, modifies the proton-donating properties of the NH_2_ group and thus the strength of hydrogen bonds in adenine quartets. According to the findings, to increase the stability of the A_4_-N3 quartet, it is recommended to introduce a strong electron-accepting substituent into each adenine molecule at the C8 or N9 positions. On the other hand, to stabilize A_4_-N7 quartet it is necessary to substitute C2 position with electron-accepting substituent or N9 position with electron-donating substituent. Substituent effect in the A_4_-N1 quartet is not pronounced and cannot be considered as a tool for tuning stability of the quartet.

The obtained results show that the electronic properties of the substituent X and aromaticity of five- and six-membered rings are affected by the tetramer formation and are slightly different depending on the A_4_ type. For C-substituted systems, the largest change in cSAR(X) values is observed for the NH_2_ group, while for the N-substituted ones the cSAR(NO_2_) changes the most. Aromaticity of both adenine rings in A_4_-N3 tetramer is more sensitive to electronic nature of the substituents than in other tetramers. Its “special properties” are most likely due to the fact that additional weak hydrogen bonds occur here as documented by the results of QTAIM analysis. In general, to increase aromaticity of the five-membered rings the electron-accepting substituents should be introduced at the C2 or C8 positions, or the electron-donating substituent at the N9 position. In turn, six-membered rings are highly aromatic (HOMA > 0.9) and slightly responsive to the substituents at the C8 position, whereas C2- and N9-substitution does not affect them.

## Conflicts of interest

There are no conflicts to declare.

## Supplementary Material

RA-010-D0RA04585C-s001
