# Supplementary material for: Substituted adenine quartets: interplay between substituent effect, hydrogen bonding, and aromaticity
Source: RSC Adv. 2020 Jun 18;10(39):23350–8. doi: 10.1039/d0ra04585c (PMC9054646; doi:10.1039/d0ra04585c)
Supplement: RA-010-D0RA04585C-s001 [file RA-010-D0RA04585C-s001.pdf]

Electronic Supplementary Material (ESI) for RSC Advances.
This journal is © The Royal Society of Chemistry 2020

Substituted adenine quartets: interplay between substituent effect, hydrogen bonding and aromaticity

Halina Szatylowicz[1,*], Paulina H. Marek[1,2], Olga A. Stasyuk[3,*], Tadeusz M. Krygowski[2], Miquel Solà[3]

[1] Warsaw University of Technology, Faculty of Chemistry, Noakowskiego 3, 00-664 Warsaw, Poland

[2] University of Warsaw, Faculty of Chemistry, Pasteura 1, 02-093 Warsaw, Poland

[3] Institute of Computational Chemistry and Catalysis and Department of Chemistry, University of Girona, C/ M. Aurèlia Capmany, 69, 17003 Girona, Spain

Electronic Supplementary Information

Table of contents

**Table S1**. Hydrogen bonds engaged in stabilizing freely optimized structures with total energy values	3ESI
**Table S2**. Interaction energy and cSAR values of analyzed systems	4ESI
**Table S3**. HOMA aromaticity index observed in the molecules of substituted adenine	5ESI
**Table S4**. cSAR values of X-substituted monomer and A_4-N1, A_4-N3, A_4-N7 systems. Given Δ values corresponds to the difference between maxima and minima in given data set	6ESI
**Figure S1**. Superposition of optimized structures of the substituted adenine tetramer depending on its type	6ESI
**Figure S2**. Comparison of cSAR(NH_2) values calculated for tetramers and monomers substituted at positions C-X and N-X	7ESI
**Table S5**. Slope values, *a*, and determination coefficients, R^2, of $cSAR_{tetramer}(NH_2)$ and $cSAR_{monomer}(NH_2)$ correlations	7ESI
**Figure S3**. Changes in cSAR(X) values depending on the substitution position and type of adenine tetramer	8ESI
**Figure S4**. Comparison of cSAR(X) values calculated for tetramers and monomers substituted at C2/C8 and N9 positions	8ESI
**Table S6**. Slope values, *a*, and determination coefficients, R^2, of $cSAR_{tetramer}(X)$ vs $cSAR_{monomer}(X)$ correlations	8ESI
**Figure S5**. Comparison of HOMA indices for both 5- and 6-membered rings calculated for tetramers substituted at the C2/C8 and N9 positions	9ESI
**Table S7**. Slope values, *a*, and determination coefficients, R^2, of $HOMA_{tetramer}(X)$ and cSAR(X) correlations for 5- and 6-membered rings	10ESI
**Figure S6**. Comparison of HOMA values for five- and six-membered rings calculated for substituted monomers and tetramers	11ESI
**Table S8**. Slope values, *a*, and determination coefficients, R^2, of $HOMA_{tetramer}(X)$ and $HOMA_{monomer}(X)$ correlations for 5- and 6-membered rings	11ESI

Cartesian coordinates of equilibrium geometries of C8-X and N9-X substituted derivatives of the A_4N1 tetramer	12ESI
Cartesian coordinates of equilibrium geometries of C8-X and N9-X substituted derivatives of the A_4N3 tetramer	19ESI
Cartesian coordinates of equilibrium geometries of C2-X and N9-X substituted derivatives of the A_4N7 tetramer	27ESI
Cartesian coordinates of equilibrium geometries substituted C2-X, C8-X and N9-X derivatives of adenine 9H	34ESI

Table S1. Hydrogen bonds engaged in stabilizing freely optimized structures with total (E_{tot}) and relative (ΔE_{rel}) energy values. $\Delta E_{rel,X}$ is calculated for each substituent X separately, $\Delta E_{rel,A4}$ – calculated by reference to A_4-N3 tetramers.

		X	DH…A	DH /Å	DA /Å	DHA /°	AH /Å	E_{tot} / kcal/mol	$\Delta E_{rel,X}$ / kcal/mol	$\Delta E_{rel,A4}$ / kcal/mol
A_4-N1	C8	NO_2	N10H···N1	1.035	2.930	160.85	1.933	-10525.41	10.04	4.61
		Cl		1.033	2.942	160.02	1.950	-9043.68	15.00	2.60
		F		1.032	2.940	160.11	1.948	-9222.11	32.71	2.02
		H		1.033	2.937	160.21	1.944	-9205.35	2.69	2.69
		Me		1.033	2.940	160.12	1.948	-10686.01	1.81	1.81
		NH_2		1.032	2.940	159.95	1.949	-10282.16	21.64	-0.15
	N9	NO_2		1.033	2.935	159.88	1.944	-10434.73	100.72	16.08
		Cl		1.032	2.944	159.67	1.954	-8930.14	128.54	7.13
		F		1.034	2.928	160.44	1.934	-9007.05	247.77	6.82
		H		1.033	2.937	160.21	1.944	-9205.35	2.69	2.69
		Me		1.033	2.939	159.79	1.948	-10655.25	32.57	3.66
		NH_2		1.033	2.932	160.17	1.939	-10171.84	131.96	2.11
A_4-N3	C8	NO_2	N10H···N3	1.030	3.039	171.15	2.017	-10530.02	5.43	**0.00**
		Cl		1.028	3.064	172.02	2.042	-9046.28	12.40	**0.00**
		F		1.028	3.061	171.89	2.040	-9224.13	30.69	**0.00**
		H		1.029	3.055	171.80	2.034	-9208.04	**0.00**	**0.00**
		Me		1.029	3.060	172.16	2.037	-10687.82	**0.00**	**0.00**
		NH_2		1.028	3.064	172.19	2.042	-10282.01	21.79	**0.00**
	N9	NO_2		1.031	3.024	167.61	2.009	-10450.81	84.64	**0.00**
		Cl		1.030	3.048	170.49	2.028	-8937.27	121.41	**0.00**
		F		1.030	3.037	170.21	2.017	-9013.87	240.95	**0.00**
		H		1.029	3.055	171.80	2.034	-9208.04	**0.00**	**0.00**
		Me		1.030	3.046	171.34	2.025	-10658.91	28.91	**0.00**
		NH_2		1.030	3.039	171.29	2.018	-10173.95	129.85	**0.00**
A_4-N7	C2	NO_2	N10H···N7	1.038	3.028	170.85	1.839	-10535.45	**0.00**	-5.43
		Cl		1.036	3.109	179.05	1.874	-9058.68	**0.00**	-12.40
		F		1.036	3.103	179.50	1.868	-9254.82	**0.00**	-30.69
		H		1.033	2.932	178.89	1.899	-9207.62	0.42	0.42
		Me		1.033	3.122	178.84	1.897	-10683.34	4.48	4.48
		NH_2		1.033	3.123	178.86	1.901	-10303.80	**0.00**	-21.79
	N9	NO_2		1.031	3.075	179.60	1.909	-10434.04	101.41	16.77
		Cl		1.032	3.108	177.85	1.902	-8930.84	127.84	6.43
		F		1.032	3.105	179.52	1.898	-9006.72	248.10	7.15
		H		1.033	2.932	178.89	1.899	-9207.62	0.42	0.42
		Me		1.034	3.120	178.93	1.889	-10658.73	29.09	0.18
		NH_2		1.034	3.108	177.20	1.891	-10173.58	130.22	0.37

Table S2. Interaction energy and cSAR values of analyzed systems. All energy values are given in kcal/mol.

		X	E_{int}	E_{prep}	E_{HB}	cSAR(X)	cSAR(NH_2)
A_4-N1	C8	NO_2	-34.55	4.58	-29.97	-0.031	0.237
		Cl	-34.54	4.34	-30.20	0.094	0.215
		F	-34.17	3.98	-30.19	0.135	0.211
		H	-34.04	4.53	-29.51	0.138	0.216
		Me	-34.33	4.68	-29.65	0.172	0.208
		NH_2	-34.16	4.12	-30.04	0.245	0.200
	N9	NO_2	-32.82	4.61	-28.21	-0.028	0.226
		Cl	-33.97	4.71	-29.26	0.030	0.220
		F	-34.23	4.50	-29.73	0.039	0.224
		H	-34.04	4.53	-29.51	0.100	0.216
		Me	-33.73	4.80	-28.93	0.126	0.210
		NH_2	-34.06	4.66	-29.40	0.115	0.216
A_4-N3	C8	NO_2	-36.28	1.70	-34.58	-0.040	0.287
		Cl	-34.37	1.57	-32.8	0.089	0.248
		F	-33.77	1.56	-32.21	0.130	0.244
		H	-33.84	1.64	-32.20	0.130	0.241
		Me	-33.10	1.64	-31.46	0.163	0.231
		NH_2	-31.77	1.88	-29.89	0.230	0.218
	N9	NO_2	-46.86	2.57	-44.29	-0.048	0.273
		Cl	-38.19	1.80	-36.39	0.021	0.260
		F	-38.41	1.86	-36.55	0.028	0.267
		H	-33.84	1.64	-32.2	0.095	0.241
		Me	-34.27	1.68	-32.59	0.123	0.236
		NH_2	-33.26	1.75	-31.51	0.114	0.232
A_4-N7	C2	NO_2	-45.97	6.88	-39.09	-0.020	0.222
		Cl	-37.37	2.65	-34.72	0.069	0.228
		F	-36.56	2.54	-34.02	0.119	0.229
		H	-34.21	2.43	-31.78	0.148	0.216
		Me	-34.14	2.40	-31.74	0.163	0.213
		NH_2	-32.77	2.57	-30.20	0.255	0.217
	N9	NO_2	-29.44	1.92	-27.52	-0.015	0.229
		Cl	-32.24	2.28	-29.96	0.042	0.222
		F	-31.48	2.08	-29.40	0.048	0.227
		H	-34.21	2.43	-31.78	0.110	0.216
		Me	-35.01	2.60	-32.41	0.138	0.211
		NH_2	-33.82	2.68	-31.14	0.124	0.216

Table S3. HOMA aromaticity index observed in the molecules of substituted adenine.

X	A_4-N1		A_4-N3		A_4-N7		monomer		
	C8-X	N9-X	C8	N9	C2	N9	C2	C8	N9
	5 ring	5 ring	5 ring	5 ring	5 ring	5 ring	5 ring	5 ring	5 ring
NO_2	0.831	0.691	0.833	0.666	0.841	0.708	0.827	0.833	0.684
Cl	0.790	0.779	0.788	0.778	0.831	0.794	0.818	0.793	0.781
F	0.762	0.822	0.758	0.826	0.830	0.837	0.814	0.764	0.825
H	0.809	0.809	0.808	0.808	0.824	0.824	0.814	0.814	0.814
Me	0.798	0.810	0.796	0.811	0.824	0.827	0.812	0.803	0.815
NH_2	0.778	0.800	0.773	0.801	0.812	0.813	0.797	0.781	0.802
range	*0.069*	*0.131*	*0.075*	*0.160*	*0.029*	*0.129*	*0.030*	*0.069*	*0.141*
average	*0.795*	*0.785*	*0.793*	*0.782*	*0.827*	*0.801*	*0.814*	*0.798*	*0.787*
SD	*0.022*	*0.044*	*0.024*	*0.054*	*0.009*	*0.043*	*0.009*	*0.022*	*0.048*

X	A_4-N1		A_4-N3		A_4-N7		monomer		
	C8-X	N9-X	C8	N9	C2	N9	C2	C8	N9
	6 ring	6 ring	6 ring	6 ring	6 ring	6 ring	6 ring	6 ring	6 ring
NO_2	0.904	0.939	0.889	0.919	0.944	0.942	0.938	0.907	0.947
Cl	0.936	0.933	0.931	0.923	0.944	0.937	0.949	0.947	0.942
F	0.942	0.926	0.938	0.917	0.941	0.932	0.945	0.953	0.935
H	0.931	0.931	0.927	0.927	0.936	0.936	0.944	0.944	0.944
Me	0.936	0.930	0.933	0.927	0.936	0.936	0.942	0.949	0.942
NH_2	0.945	0.934	0.942	0.931	0.928	0.938	0.937	0.957	0.947
range	*0.041*	*0.013*	*0.053*	*0.014*	*0.016*	*0.010*	*0.012*	*0.050*	*0.012*
average	*0.932*	*0.932*	*0.927*	*0.924*	*0.938*	*0.937*	*0.943*	*0.943*	*0.943*
SD	*0.013*	*0.004*	*0.018*	*0.005*	*0.006*	*0.003*	*0.004*	*0.017*	*0.004*

Table S4. cSAR values of X-substituted monomer and A_4-N1, A_4-N3, A_4-N7 systems. Given Δ values corresponds to the difference between maxima and minima in given data set.

	**Monomer**		**A_4-N1**	**A_4-N3**	**A_4-N7**	**Δ**
**X**	C2-X	C8-X	C8-X	C8-X	C2-X	
NO_2	-0.020	-0.045	-0.031	-0.040	-0.020	0.025
Cl	0.075	0.094	0.094	0.089	0.069	0.025
F	0.124	0.135	0.135	0.130	0.119	0.016
H	0.151	0.140	0.138	0.130	0.148	0.021
Me	0.171	0.173	0.172	0.163	0.163	0.010
NH_2	0.268	0.247	0.245	0.230	0.255	0.038
**Δ**	0.288	0.292	0.276	0.270	0.275	

	**Monomer**	**A_4-N1**	**A_4-N3**	**A_4-N7**	**Δ**
**X**		N9-X			
NO_2	-0.038	-0.028	-0.048	-0.015	0.033
Cl	0.029	0.030	0.021	0.042	0.021
F	0.036	0.039	0.028	0.048	0.020
H	0.101	0.100	0.095	0.110	0.015
Me	0.129	0.126	0.123	0.138	0.015
NH_2	0.117	0.115	0.114	0.124	0.010
**Δ**	0.167	0.154	0.171	0.153	

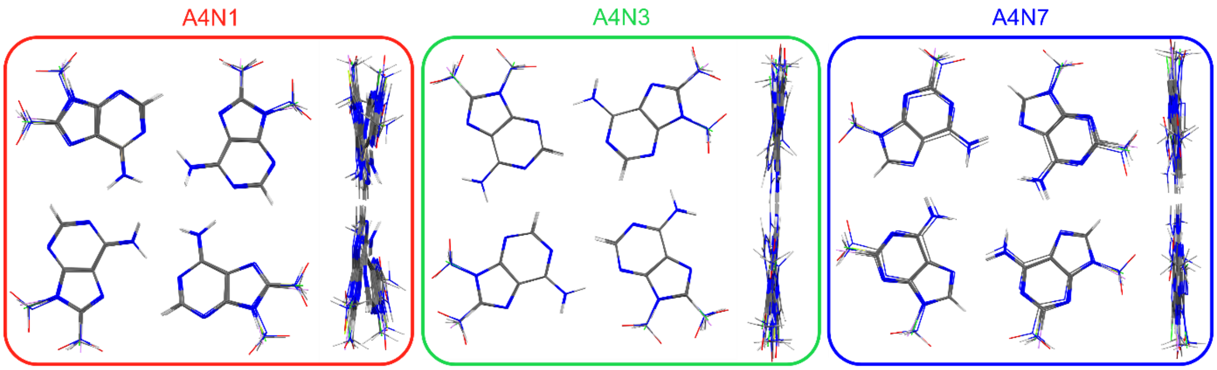

Figure S1. Superposition of optimized structures of the substituted adenine tetramer depending on its type.

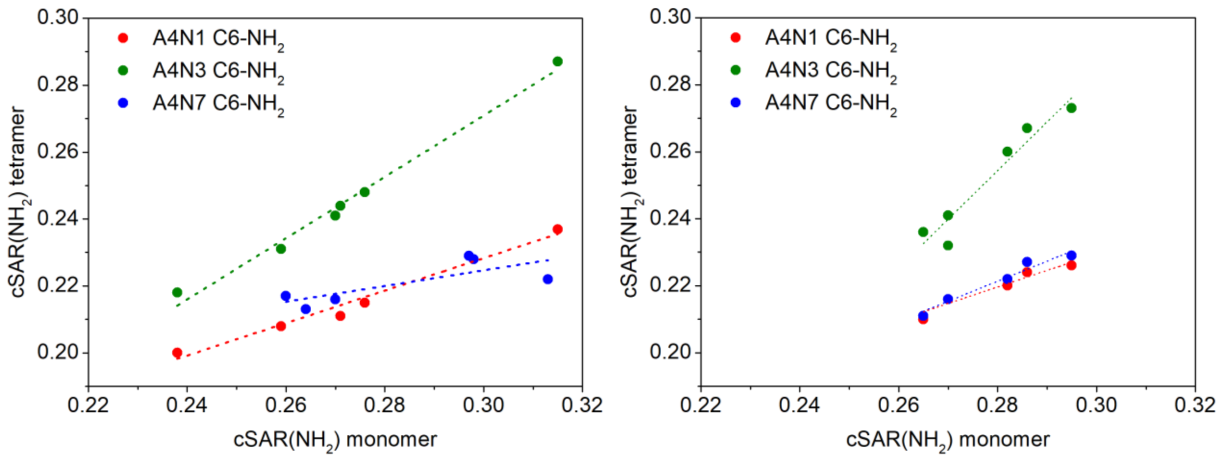

Figure S2. Comparison of cSAR(NH_2) values calculated for tetramers and monomers substituted at positions C-X (a) and N-X (b).

Table S5. Slope values, *a*, and determination coefficients, R^2, of $cSAR_{tetramer}(NH_2)$ and $cSAR_{monomer}(NH_2)$ correlations.

Substitution position	C2/C8		N9	
Quartet type	*a*	R^2	*a*	R^2
A_4-N1	0.485	0.969	0.492	0.932
A_4-N3	0.916	0.988	1.449	0.929
A_4-N7	0.234	0.596	0.598	0.964

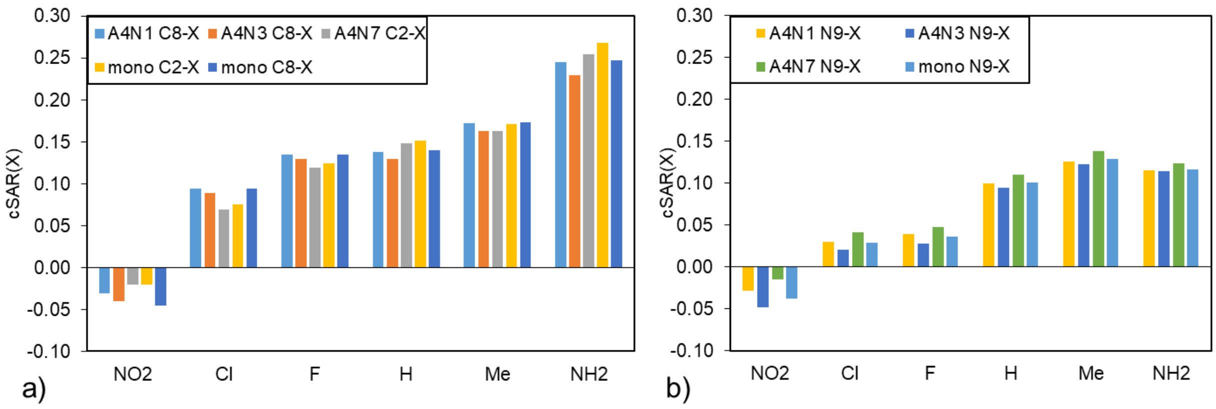

Figure S3. Changes in cSAR(X) values depending on the substitution position and type of adenine tetramer.

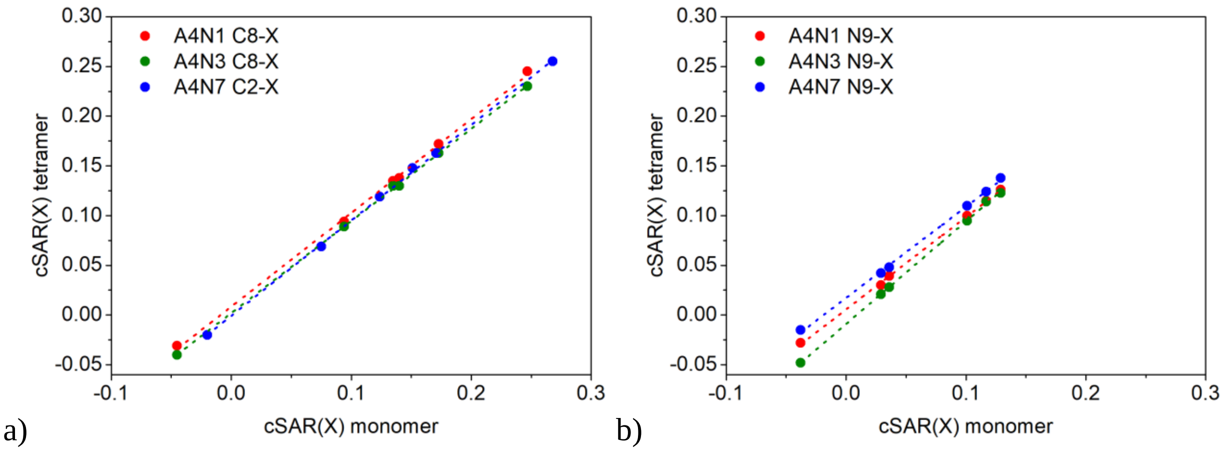

Figure S4. Comparison of cSAR(X) values calculated for tetramers and monomers substituted at C2/C8 (a) and N9 (b) positions.

Table S6. Slope values, *a*, and determination coefficients, R^2, of $cSAR_{tetramer}(X)$ vs $cSAR_{monomer}(X)$ correlations.

	C2/C8		N9	
	$cSAR_{tetramer}(X)$ *vs* $cSAR_{monomer}(X)$			
	a	R^2	*A*	R^2
A_4-N1	0.943	0.999	0.930	0.999
A_4-N3	0.926	1.000	1.033	1.000
A_4-N7	0.960	0.999	0.917	0.999

a) A_4N1 substituted derivatives

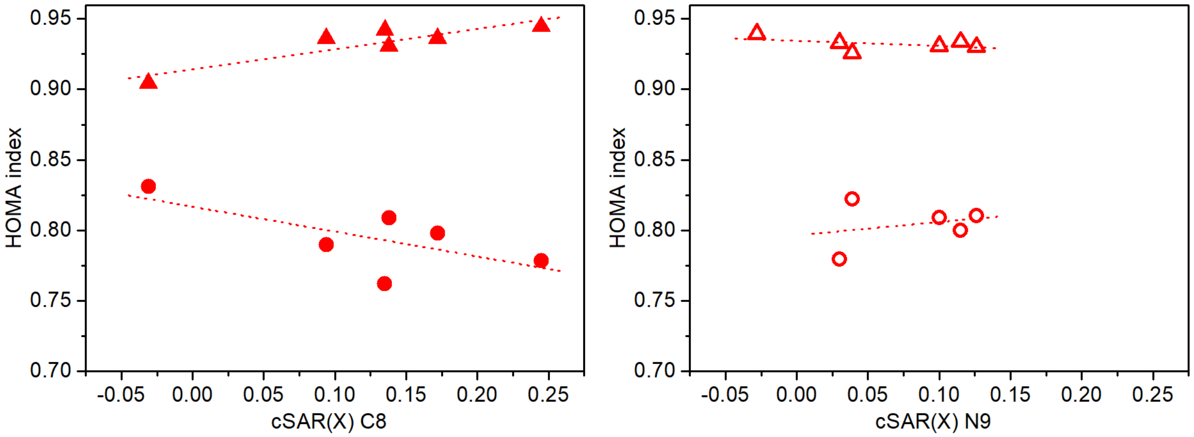

b) A_4N3 substituted derivatives

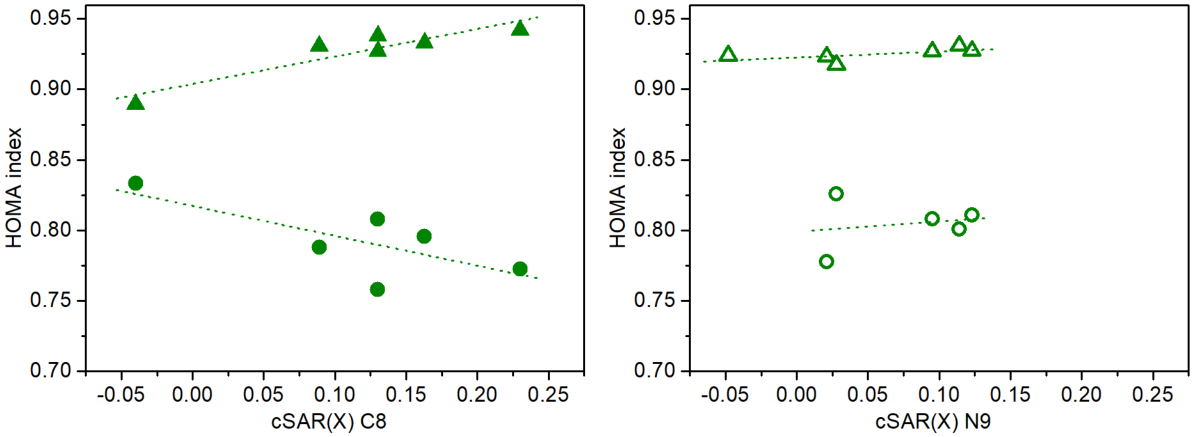

c) A_4N7 substituted derivatives

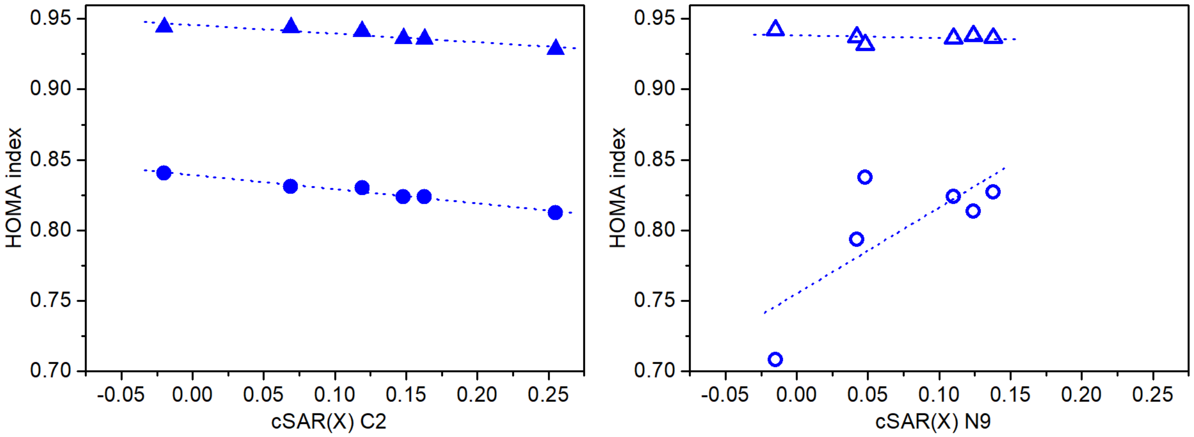

Figure S5. Comparison of HOMA indices for both 5- and 6-membered rings (denoted by circles and triangles, respectively) calculated for tetramers substituted at the C2/C8 and N9 positions.

Table S7. Slope values, *a*, and determination coefficients, R^2, of $HOMA_{tetramer}(X)$ and cSAR(X) correlations for 5- and 6-membered rings.

Substitution position	C2/C8		N9	
Quartet type	*a*	R^2	*a*	R^2
	5-membered rings			
A_4-N1	-0.177	0.453	0.618	0.587
A_4-N3	-0.212	0.516	0.678	0.588
A_4-N7	-0.101	0.971	0.609	0.573
	6-membered rings			
A_4-N1	0.142	0.804	-0.035	0.220
A_4-N3	0.195	0.853	0.043	0.382
A_4-N7	-0.061	0.874	-0.019	0.117

(a) For five-membered rings

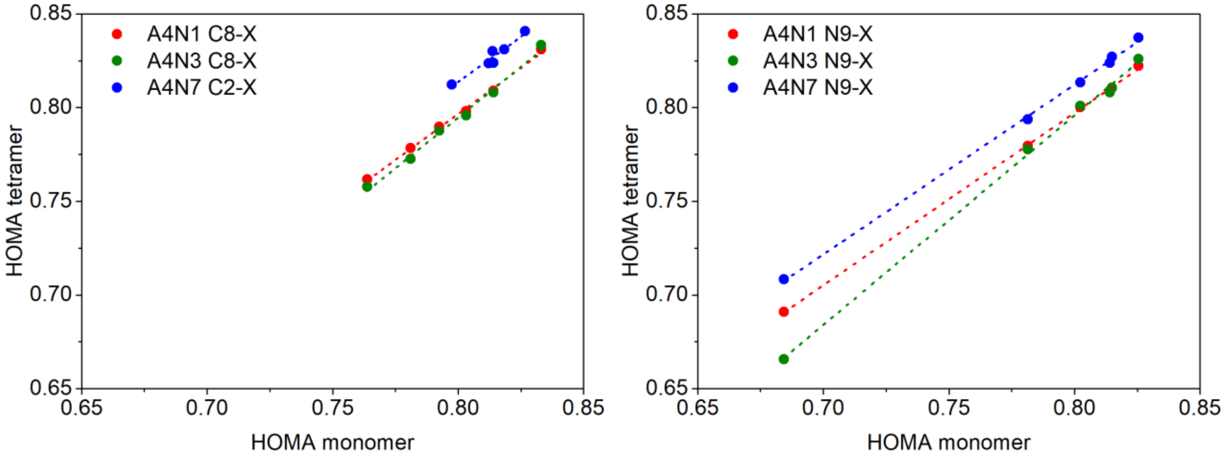

(b) For six-membered rings

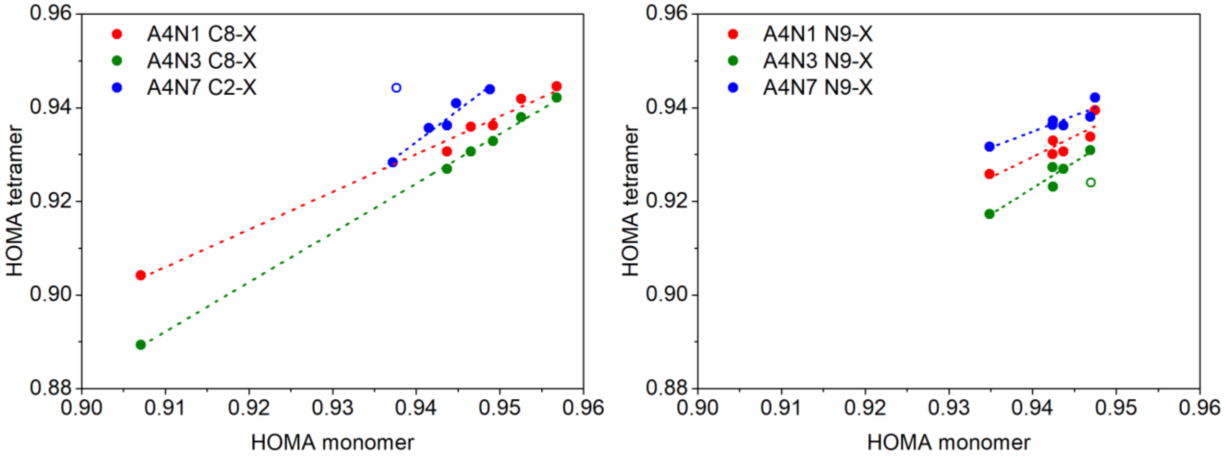

Figure S6. Comparison of HOMA values for five- and six-membered rings calculated for substituted monomers and tetramers.

Table S8. Slope values, *a*, and determination coefficients, R^2, of $HOMA_{tetramer}(X)$ and $HOMA_{monomer}(X)$ correlations for 5- and 6-membered rings.

Substitution position	C2/C8		N9	
Quartet type	*a*	R^2	*a*	R^2
	5-membered rings			
A_4-N1	0.981	0.997	0.921	0.999
A_4-N3	1.081	0.993	1.119	0.998
A_4-N7	0.960	0.939	0.906	0.999
	6-membered rings			
A_4-N1	0.805	0.989	0.879	0.771
A_4-N3	1.056	0.999	1.115	0.914
A_4-N7	1.353	0.950	0.683	0.847

Cartesian coordinates of equilibrium geometries of C8-NO_2 and N9-NO_2 substituted derivatives of the A_4N1 tetramer

	C8-NO_2				N9-NO_2		
N	-3.635420	-0.530605	-0.006233	N	-3.648254	-0.530091	-0.071077
C	-4.962249	-0.557903	0.257157	C	-4.977806	-0.534344	0.138687
N	-5.730011	-1.626533	0.524056	N	-5.773258	-1.589663	0.388399
C	-5.019508	-2.762324	0.515117	C	-5.079992	-2.731352	0.422517
C	-3.631362	-2.890810	0.261110	C	-3.696038	-2.886039	0.230084
C	-2.930845	-1.689348	-0.026578	C	-2.964712	-1.705553	-0.043709
N	-5.454484	-4.056275	0.718891	N	-5.520588	-4.056204	0.624164
C	-4.338510	-4.857366	0.573890	C	-4.396228	-4.895082	0.537112
N	-3.229383	-4.208366	0.298868	N	-3.299434	-4.223300	0.303322
H	-5.454256	0.413926	0.246774	H	-5.456546	0.443155	0.102364
N	-1.613000	-1.664396	-0.317042	H	-4.496219	-5.964862	0.657476
H	-1.016185	-2.507351	-0.257681	N	-1.632777	-1.694597	-0.273941
H	-1.233797	-0.819941	-0.726861	H	-1.043568	-2.539025	-0.195621
H	-6.394215	-4.383130	0.922762	H	-1.236520	-0.863975	-0.695379
N	-0.530456	3.634841	0.010795	N	-0.530179	3.648349	0.071874
C	-0.556475	4.961044	-0.255638	C	-0.534249	4.977759	-0.138646
N	-1.624306	5.728930	-0.525243	N	-1.589415	5.773125	-0.389112
C	-2.760670	5.019338	-0.515769	C	-2.731149	5.079926	-0.423255
C	-2.890430	3.631912	-0.258736	C	-2.886020	3.696109	-0.230089
C	-1.689749	2.931205	0.031686	C	-1.705684	2.964870	0.044581
N	-4.054125	5.454818	-0.721692	N	-4.055898	5.520494	-0.625635
C	-4.856219	4.339839	-0.574621	C	-4.894904	4.396263	-0.538193
N	-4.208282	3.230891	-0.296639	N	-4.223291	3.299570	-0.303584
H	0.415752	5.452281	-0.245518	H	0.443276	5.456442	-0.102312
N	-1.666043	1.614042	0.325379	H	-5.964635	4.496266	-0.658960
H	-0.822422	1.235179	0.737160	N	-1.694910	1.633094	0.275780
H	-2.509095	1.017504	0.265602	H	-0.864374	1.237036	0.697592
H	-4.380101	6.394284	-0.928118	H	-2.539251	1.043798	0.197247
N	3.635589	0.530755	-0.007453	N	3.648241	0.530099	-0.070742
C	4.962467	0.558459	0.255245	C	4.977781	0.534343	0.139073
N	5.730154	1.627397	0.520961	N	5.773219	1.589654	0.388793
C	5.019505	2.763094	0.511621	C	5.079953	2.731343	0.422897
C	3.631274	2.891176	0.258159	C	3.696011	2.886041	0.230412
C	2.930870	1.689405	-0.028431	C	2.964695	1.705560	-0.043439
N	5.454348	4.057277	0.714443	N	5.520539	4.056188	0.624571
C	4.338200	4.858090	0.569321	C	4.396193	4.895077	0.537497
N	3.229106	4.208691	0.295180	N	3.299409	4.223304	0.303643
H	5.454614	-0.413301	0.245354	H	5.456522	-0.443156	0.102778
N	1.613032	1.664191	-0.318631	H	4.496184	5.964856	0.657890
H	1.016149	2.507043	-0.259227	N	1.632766	1.694598	-0.273803
H	1.233601	0.819242	-0.727097	H	1.043551	2.539030	-0.195564
H	6.394070	4.384425	0.917884	H	1.236595	0.863993	-0.695328
N	0.530776	-3.634984	0.012025	N	0.530153	-3.648291	0.072108
C	0.556564	-4.961268	-0.254190	C	0.534257	-4.977734	-0.138447
N	1.624192	-5.729332	-0.524006	N	1.589456	-5.773135	-0.388855
C	2.760614	-5.019828	-0.515090	C	2.731191	-5.079938	-0.422828
C	2.890618	-3.632337	-0.258408	C	2.886033	-3.696098	-0.229653
C	1.690139	-2.931436	0.032265	C	1.705661	-2.964823	0.044847
N	4.053927	-5.455495	-0.721391	N	4.055959	-5.520522	-0.624974
C	4.856202	-4.340562	-0.575071	C	4.894954	-4.396283	-0.537532
N	4.208495	-3.231457	-0.296871	N	4.223312	-3.299566	-0.303008
H	-0.415689	-5.452440	-0.243668	H	-0.443271	-5.456418	-0.102200

N	1.666643	-1.614198	0.325562	H	5.964703	-4.496299	-0.658197
H	0.823150	-1.235107	0.737355	N	1.694847	-1.633010	0.275855
H	2.509656	-1.017651	0.265211	H	0.864335	-1.236915	0.697666
H	4.379713	-6.395055	-0.927604	H	2.539233	-1.043767	0.197416
N	-4.454652	-6.303738	0.718643	N	-6.891377	-4.541066	0.868247
O	-5.601886	-6.729557	0.984342	O	-6.984530	-5.761619	0.974582
O	-3.435990	-6.988380	0.570549	O	-7.756540	-3.686836	0.934587
N	-6.302405	4.456801	-0.720684	N	-4.540553	6.891157	-0.870786
O	-6.726929	5.603467	-0.991269	O	-5.761057	6.984348	-0.977643
O	-6.988104	3.439341	-0.568906	O	-3.686220	7.756194	-0.937349
N	4.454102	6.304570	0.713096	N	6.891328	4.541033	0.868627
O	5.601394	6.730822	0.978422	O	6.984496	5.761580	0.974926
O	3.435181	6.988883	0.564639	O	7.756472	3.686789	0.934961
N	6.302325	-4.457763	-0.721929	N	4.540645	-6.891211	-0.869840
O	6.726629	-5.604602	-0.991973	O	3.686317	-7.756250	-0.936459
O	6.988205	-3.440322	-0.571264	O	5.761163	-6.984415	-0.976414

Cartesian coordinates of equilibrium geometries of C8-Cl and N9-Cl substituted derivatives of the A_4N1 tetramer

	C8-Cl				N9-Cl		
N	-3.648263	-0.584644	-0.028330	N	-3.645809	-0.585960	-0.116480
C	-4.977293	-0.599049	0.198737	C	-4.984195	-0.598053	0.054686
N	-5.756161	-1.660967	0.464962	N	-5.776709	-1.658819	0.274837
C	-5.043417	-2.796659	0.493841	C	-5.071004	-2.795243	0.324536
C	-3.659273	-2.938300	0.282205	C	-3.679902	-2.945289	0.177903
C	-2.946691	-1.749906	-0.005270	C	-2.953089	-1.754591	-0.072472
N	-5.498065	-4.090265	0.710572	N	-5.519655	-4.099825	0.505986
C	-4.384305	-4.911008	0.612745	C	-4.410914	-4.944540	0.460401
N	-3.269379	-4.276046	0.359032	N	-3.293497	-4.280758	0.262870
H	-5.463618	0.374778	0.160159	H	-5.464759	0.378241	0.009314
N	-1.616281	-1.718180	-0.252009	H	-4.522206	-6.014969	0.577976
H	-1.013510	-2.554189	-0.188702	N	-1.614337	-1.729459	-0.267460
H	-1.238494	-0.880966	-0.677497	H	-1.014339	-2.563918	-0.172330
H	-6.453645	-4.377116	0.889954	H	-1.216995	-0.892774	-0.674850
N	-0.584722	3.648554	0.034162	N	-0.585911	3.646144	0.117485
C	-0.598575	4.977085	-0.195573	C	-0.598105	4.984513	-0.053408
N	-1.660042	5.755610	-0.464724	N	-1.658918	5.776891	-0.273777
C	-2.795866	5.043077	-0.493688	C	-2.795260	5.071062	-0.324052
C	-2.938001	3.659368	-0.279612	C	-2.945187	3.679942	-0.177374
C	-1.750089	2.947155	0.010984	C	-1.754462	2.953300	0.073313
N	-4.089176	5.497601	-0.712675	N	-4.099836	5.519574	-0.506461
C	-4.910267	4.384237	-0.613360	C	-4.944454	4.410745	-0.461133
N	-4.275785	3.269682	-0.357220	N	-4.280594	3.293407	-0.262836
H	0.375331	5.463261	-0.156739	H	0.378138	5.465166	-0.007579
N	-1.718905	1.617245	0.260755	H	-6.014869	4.521927	-0.579506
H	-0.882463	1.240317	0.688687	N	-1.729105	1.614574	0.268319
H	-2.554716	1.014265	0.196585	H	-0.891914	1.217447	0.675758
H	-4.375683	6.452947	-0.893957	H	-2.563599	1.014420	0.174232
N	3.648333	0.584635	-0.030498	N	3.646177	0.586107	-0.119756
C	4.977392	0.598968	0.196109	C	4.984513	0.598121	0.051558
N	5.756234	1.660742	0.462555	N	5.776751	1.658634	0.273489
C	5.043405	2.796351	0.492400	C	5.070805	2.794819	0.325401
C	3.659223	2.938063	0.281510	C	3.679741	2.944939	0.178898
C	2.946667	1.749823	-0.006570	C	2.953258	1.754541	-0.073966
N	5.497995	4.089844	0.709485	N	5.519112	4.099119	0.509584

C	4.384183	4.910575	0.612744	C	4.410256	4.943726	0.465529
N	3.269247	4.275730	0.359315	N	3.293060	4.280163	0.266730
H	5.463788	-0.374790	0.156815	H	5.465263	-0.377997	0.004638
N	1.616203	1.718193	-0.253207	H	4.521272	6.013901	0.585349
H	1.013448	2.554137	-0.189030	N	1.614560	1.729330	-0.269501
H	1.238520	0.881442	-0.679311	H	1.014425	2.563799	-0.174077
H	6.453573	4.376635	0.888403	H	1.217900	0.893610	-0.678961
N	0.585119	-3.648388	0.032315	N	0.586268	-3.646056	0.119676
C	0.598592	-4.976807	-0.198182	C	0.598232	-4.984346	-0.051834
N	1.659656	-5.755250	-0.468951	N	1.658724	-5.776580	-0.274047
C	2.795484	-5.042770	-0.498836	C	2.794982	-5.070713	-0.325972
C	2.937990	-3.659182	-0.284346	C	2.945127	-3.679686	-0.179264
C	1.750483	-2.947051	0.008072	C	1.754734	-2.953174	0.073786
N	4.088483	-5.497218	-0.719294	N	4.099311	-5.519054	-0.510379
C	4.909794	-4.383982	-0.620770	C	4.943971	-4.410249	-0.466056
N	4.275698	-3.269526	-0.363427	N	4.280397	-3.293065	-0.267043
H	-0.375292	-5.462940	-0.158524	H	-0.377921	-5.465062	-0.004889
N	1.719670	-1.617201	0.258475	H	6.014188	-4.521301	-0.585853
H	0.884063	-1.240675	0.688247	N	1.729684	-1.614474	0.269387
H	2.555582	-1.014467	0.194037	H	0.894136	-1.217564	0.678946
H	4.374701	-6.452454	-0.900923	H	2.564061	-1.014466	0.173146
Cl	-4.547908	-6.624144	0.815277	Cl	-7.162491	-4.584595	0.720944
Cl	-6.623203	4.547842	-0.817220	Cl	-4.584702	7.162366	-0.722142
Cl	4.547730	6.623607	0.816064	Cl	7.161763	4.583739	0.725895
Cl	6.622501	-4.547633	-0.826759	Cl	4.583931	-7.161687	-0.727112

Cartesian coordinates of equilibrium geometries of C8-F and N9-F substituted derivatives of the A_4N1 tetramer

		C8-F				N9-F	
N	-3.650180	-0.585492	-0.023712	N	-3.637809	-0.560003	-0.026500
C	-4.976010	-0.596408	0.213198	C	-4.963120	-0.569520	0.228999
N	-5.747942	-1.653438	0.521200	N	-5.742431	-1.624834	0.511586
C	-5.029592	-2.781762	0.583571	C	-5.029260	-2.759028	0.525055
C	-3.647169	-2.926014	0.365662	C	-3.648693	-2.917816	0.285274
C	-2.942972	-1.747303	0.032852	C	-2.941460	-1.726821	-0.016971
N	-5.478830	-4.073851	0.854831	N	-5.429301	-4.063070	0.744251
C	-4.357852	-4.872672	0.776066	C	-4.352815	-4.921921	0.636926
N	-3.248231	-4.264748	0.491249	N	-3.252594	-4.251016	0.355009
H	-5.467730	0.372963	0.145860	H	-5.444740	0.406911	0.201397
N	-1.612829	-1.716188	-0.221127	H	-4.463149	-5.989637	0.773309
H	-1.012160	-2.553378	-0.156558	N	-1.618379	-1.703761	-0.291654
H	-1.243557	-0.894068	-0.682370	H	-1.015265	-2.540166	-0.220549
H	-6.428988	-4.365556	1.053915	H	-1.239710	-0.864707	-0.713234
N	-0.585894	3.651350	0.030937	N	-0.562285	3.638909	0.026038
C	-0.597260	4.976986	-0.206931	C	-0.574283	4.965983	-0.221488
N	-1.654395	5.748206	-0.516028	N	-1.631271	5.746071	-0.496349
C	-2.782340	5.029290	-0.578633	C	-2.764547	5.031539	-0.510549
C	-2.926133	3.646968	-0.359822	C	-2.920948	3.649202	-0.278799
C	-1.747340	2.943560	-0.025727	C	-1.728288	2.941159	0.015630
N	-4.074420	5.477742	-0.851086	N	-4.069835	5.431595	-0.722287
C	-4.872802	4.356473	-0.772190	C	-4.926838	4.353224	-0.618582
N	-4.264590	3.247326	-0.485892	N	-4.253843	3.251792	-0.346515
H	0.371812	5.469248	-0.139383	H	0.401452	5.448965	-0.193388
N	-1.715849	1.613638	0.229592	H	-5.995023	4.463103	-0.750089
H	-0.893746	1.245161	0.691396	N	-1.703464	1.616213	0.282565

H	-2.552614	1.012391	0.164137	H	-0.863390	1.236461	0.699695
H	-4.366396	6.427605	-1.050984	H	-2.540318	1.013823	0.212634
N	3.651116	0.586017	-0.029123	N	3.636618	0.560203	-0.037094
C	4.977143	0.596598	0.206874	C	4.963785	0.567629	0.208730
N	5.749097	1.652937	0.516854	N	5.746525	1.621504	0.487060
C	5.030521	2.780915	0.582499	C	5.034951	2.756638	0.507113
C	3.647909	2.925465	0.365768	C	3.652844	2.917435	0.277589
C	2.943678	1.747490	0.030595	C	2.941894	1.727944	-0.021227
N	5.479639	4.072305	0.856937	N	5.438095	4.059757	0.725585
C	4.358416	4.871091	0.781179	C	4.361662	4.919963	0.627507
N	3.248728	4.263727	0.494954	N	3.258728	4.250868	0.352508
H	5.469062	-0.372457	0.136797	H	5.443793	-0.409457	0.176517
N	1.613227	1.716737	-0.222585	H	4.474194	5.987229	0.764706
H	1.012768	2.553958	-0.156514	N	1.616762	1.706920	-0.286382
H	1.244240	0.895829	-0.686161	H	1.015401	2.544275	-0.213190
H	6.429820	4.363631	1.056311	H	1.234602	0.869006	-0.706341
N	0.586276	-3.650830	0.026931	N	0.559266	-3.636815	0.020020
C	0.596649	-4.976346	-0.212082	C	0.570135	-4.963874	-0.225532
N	1.652717	-5.747578	-0.525398	N	1.626535	-5.744660	-0.499280
C	2.780555	-5.028792	-0.590955	C	2.760387	-5.030980	-0.515076
C	2.925227	-3.646576	-0.371592	C	2.917812	-3.648655	-0.285351
C	1.747614	-2.943196	-0.033201	C	1.725798	-2.939956	0.008953
N	4.071649	-5.477251	-0.867828	N	4.065282	-5.431917	-0.727364
C	4.870421	-4.356108	-0.790664	C	4.923207	-4.354165	-0.625711
N	4.263311	-3.247052	-0.501841	N	4.250977	-3.252267	-0.354294
H	-0.372327	-5.468434	-0.141771	H	-0.406082	-5.445867	-0.196926
N	1.717035	-1.613260	0.222484	H	5.991314	-4.464903	-0.757972
H	0.897267	-1.245296	0.688989	N	1.701756	-1.615160	0.275088
H	2.554198	-1.012710	0.156395	H	0.861467	-1.234506	0.692289
H	4.362821	-6.427027	-1.069425	H	2.538370	-1.012592	0.203358
F	-4.489531	-6.188100	0.990982	F	-6.732721	-4.463672	1.033188
F	-6.188140	4.487410	-0.988402	F	-4.473061	6.736463	-1.001546
F	4.489938	6.185928	1.000060	F	6.743897	4.458160	1.006888
F	6.184967	-4.487050	-1.011090	F	4.467413	-6.737200	-1.005231

Cartesian coordinates of equilibrium geometries of C8-H and N9-H substituted derivatives of the A_4N1 tetramer

	C8-H / N9-H		
N	-3.639812	-0.589874	-0.054963
C	-4.970931	-0.602642	0.165863
N	-5.753605	-1.661755	0.425273
C	-5.042533	-2.802412	0.455918
C	-3.656905	-2.946799	0.251483
C	-2.941979	-1.756547	-0.029985
N	-5.499071	-4.091715	0.668243
C	-4.394581	-4.928699	0.581263
N	-3.273669	-4.282532	0.331237
H	-5.452303	0.373861	0.128217
H	-4.485339	-6.000837	0.709546
N	-1.611224	-1.727027	-0.271989
H	-1.004210	-2.558453	-0.188432
H	-1.225954	-0.883187	-0.676968
N	-0.590567	3.641156	0.063624
C	-0.601809	4.971309	-0.162640
N	-1.659585	5.753470	-0.428715

C	-2.800531	5.042893	-0.460657
C	-2.946384	3.658162	-0.251481
C	-1.757574	2.943837	0.037516
N	-4.088715	5.499131	-0.679469
C	-4.926633	4.395409	-0.591337
N	-4.281991	3.275308	-0.335104
H	0.374904	5.452176	-0.123797
H	-5.998201	4.486126	-0.723820
N	-1.729516	1.614302	0.286106
H	-0.886593	1.230146	0.693416
H	-2.560162	1.006627	0.200304
N	3.639435	0.590376	-0.062659
C	4.969987	0.601704	0.162297
N	5.752309	1.659330	0.428329
C	5.041568	2.800135	0.461575
C	3.656553	2.945937	0.253549
C	2.941894	1.757180	-0.034992
N	5.497889	4.088191	0.681120
C	4.393898	4.925988	0.594449
N	3.273567	4.281423	0.338280
H	5.451017	-0.374851	0.122443
H	4.484650	5.997470	0.727714
N	1.611767	1.729070	-0.281171
H	1.004348	2.559815	-0.193980
H	1.227104	0.886273	-0.688333
N	0.591319	-3.640672	0.064046
C	0.602645	-4.970821	-0.162553
N	1.660292	-5.752692	-0.430299
C	2.801012	-5.041820	-0.463604
C	2.946702	-3.657017	-0.254350
C	1.758079	-2.943013	0.036314
N	4.089114	-5.497795	-0.684123
C	4.926839	-4.393867	-0.596544
N	4.282153	-3.273865	-0.339242
H	-0.373884	-5.451961	-0.122620
H	5.998330	-4.484359	-0.730181
N	1.730088	-1.613463	0.284813
H	0.887751	-1.229240	0.693991
H	2.560130	-1.005410	0.196135
H	-6.461128	-4.356163	0.846456
H	-4.351945	6.460588	-0.862106
H	6.459502	4.351396	0.863560
H	4.352487	-6.459302	-0.866967

Cartesian coordinates of equilibrium geometries of C8-Me and N9-Me substituted derivatives of the A_4N1 tetramer

	C8-Me				N9-Me		
N	-3.644380	-0.571956	-0.037863	N	-3.638436	-0.603671	-0.129354
C	-4.973557	-0.583921	0.185620	C	-4.978134	-0.617973	0.032393
N	-5.760929	-1.646917	0.420562	N	-5.770884	-1.679897	0.246496
C	-5.055213	-2.790679	0.420106	C	-5.059793	-2.821257	0.303314
C	-3.671559	-2.935742	0.210175	C	-3.665068	-2.962123	0.167090
C	-2.952302	-1.743840	-0.042480	C	-2.940962	-1.770284	-0.079889
N	-5.515977	-4.083958	0.596433	N	-5.539876	-4.108201	0.481397
C	-4.417707	-4.936722	0.483389	C	-4.426967	-4.938956	0.443483

N	-3.297276	-4.276122	0.251330	N	-3.289035	-4.297106	0.256738
H	-5.450738	0.395330	0.173115	H	-5.457104	0.359277	-0.017114
N	-1.620722	-1.713296	-0.286959	H	-4.529481	-6.012116	0.559066
H	-1.017372	-2.548611	-0.220781	N	-1.600798	-1.736449	-0.268474
H	-1.234687	-0.864129	-0.679391	H	-0.995572	-2.567084	-0.168306
H	-6.480371	-4.346471	0.763981	H	-1.205502	-0.895690	-0.669603
N	-0.571907	3.644205	0.038059	N	-0.603675	3.638509	0.130865
C	-0.583679	4.973383	-0.185417	C	-0.617880	4.978135	-0.031884
N	-1.646584	5.760911	-0.420116	N	-1.679735	5.770833	-0.247172
C	-2.790458	5.055375	-0.419568	C	-2.821111	5.059765	-0.303790
C	-2.935721	3.671740	-0.209735	C	-2.962058	3.665105	-0.166679
C	-1.743895	2.952309	0.042758	C	-1.770310	2.941080	0.081212
N	-4.083689	5.516353	-0.595686	N	-4.107976	5.539820	-0.482376
C	-4.936628	4.418235	-0.482628	C	-4.938789	4.426962	-0.444175
N	-4.276175	3.297689	-0.250782	N	-4.297037	3.289091	-0.256570
H	0.395644	5.450416	-0.173120	H	0.359398	5.457067	0.017780
N	-1.713451	1.620741	0.287110	H	-6.011919	4.529473	-0.560166
H	-0.864326	1.234502	0.679490	N	-1.736501	1.601030	0.270381
H	-2.548783	1.017358	0.220873	H	-0.895875	1.205721	0.671938
H	-4.346067	6.480787	-0.763136	H	-2.567027	0.995576	0.169845
N	3.644037	0.571797	-0.037772	N	3.638209	0.603700	-0.129452
C	4.973258	0.583622	0.185516	C	4.977938	0.617997	0.032514
N	5.760834	1.646567	0.420007	N	5.770757	1.679920	0.246915
C	5.055290	2.790437	0.419296	C	5.059704	2.821297	0.303480
C	3.671616	2.935647	0.209514	C	3.664969	2.962174	0.167056
C	2.952133	1.743785	-0.042646	C	2.940790	1.770340	-0.080005
N	5.516284	4.083699	0.595203	N	5.539816	4.108229	0.481282
C	4.418114	4.936590	0.482013	C	4.426907	4.938985	0.443412
N	3.297534	4.276093	0.250276	N	3.288949	4.297160	0.256708
H	5.450294	-0.395702	0.173236	H	5.456880	-0.359274	-0.017102
N	1.620520	1.713391	-0.286928	H	4.529440	6.012144	0.558928
H	1.017325	2.548761	-0.220908	N	1.600597	1.736563	-0.268687
H	1.234328	0.864102	-0.678925	H	0.995507	2.567249	-0.168784
H	6.480748	4.346123	0.762617	H	1.205406	0.895705	-0.669795
N	0.571796	-3.644216	0.038110	N	0.603801	-3.638412	0.130781
C	0.583571	-4.973342	-0.185539	C	0.617962	-4.978054	-0.031954
N	1.646462	-5.760791	-0.420438	N	1.679768	-5.770820	-0.247115
C	2.790322	-5.055227	-0.419922	C	2.821165	-5.059801	-0.303697
C	2.935568	-3.671633	-0.209816	C	2.962164	-3.665142	-0.166585
C	1.743764	-2.952284	0.042921	C	1.770466	-2.941035	0.081315
N	4.083546	-5.516125	-0.596393	N	4.108011	-5.539903	-0.482191
C	4.936459	-4.417992	-0.483170	C	4.938846	-4.427063	-0.444038
N	4.275996	-3.297521	-0.250882	N	4.297146	-3.289161	-0.256456
H	-0.395740	-5.450398	-0.173217	H	-0.359322	-5.456962	0.017650
N	1.713381	-1.620756	0.287689	H	6.011965	-4.529603	-0.560022
H	0.864236	-1.234735	0.680187	N	1.736837	-1.600960	0.270791
H	2.548708	-1.017451	0.221523	H	0.896178	-1.205897	0.672444
H	4.345940	-6.480521	-0.764142	H	2.567446	-0.995803	0.170577
C	-4.549363	-6.421900	0.607376	C	-6.943848	-4.483553	0.641927
H	-4.977411	-6.705798	1.577989	H	-7.362737	-4.005944	1.533574
H	-5.199872	-6.829895	-0.178268	H	-7.520475	-4.160886	-0.231240
H	-3.559823	-6.875535	0.512707	H	-7.010863	-5.569764	0.743234
C	-6.421806	4.550197	-0.606314	C	-4.483213	6.943736	-0.643525
H	-6.829599	5.200146	0.179901	H	-4.160746	7.520676	0.229487
H	-6.705777	4.979066	-1.576538	H	-4.005365	7.362279	-1.535187
H	-6.875587	3.560662	-0.512349	H	-5.569379	7.010733	-0.745125

C	4.550036	6.421786	0.605469	C	6.943787	4.483578	0.641531
H	4.978946	6.705917	1.575647	H	7.362848	4.006101	1.533129
H	5.199934	6.829471	-0.180855	H	7.520271	4.160821	-0.231657
H	3.560461	6.875511	0.511487	H	7.010761	5.569776	0.742678
C	6.421629	-4.549839	-0.607176	C	4.483227	-6.943821	-0.643266
H	6.829596	-5.200244	0.178620	H	4.160767	-7.520707	0.229773
H	6.705468	-4.978129	-1.577731	H	4.005383	-7.362421	-1.534891
H	6.875355	-3.560307	-0.512712	H	5.569388	-7.010814	-0.744859

Cartesian coordinates of equilibrium geometries of C8-NH_2 and N9-NH_2 substituted derivatives of the A_4N1 tetramer

		C8-NH_2				N9-NH_2	
N	-3.637548	-0.549606	-0.087614	N	-3.636071	-0.659311	-0.032192
C	-4.924688	-0.509278	0.300703	C	-4.962142	-0.688000	0.210492
N	-5.683690	-1.531547	0.738387	N	-5.723745	-1.759054	0.492025
C	-4.993985	-2.680804	0.756244	C	-4.989381	-2.883069	0.521791
C	-3.652402	-2.877484	0.384880	C	-3.606808	-3.013453	0.300422
C	-2.961423	-1.731737	-0.066038	C	-2.917630	-1.814967	-0.007329
N	-5.445930	-3.944547	1.127282	N	-5.427894	-4.181013	0.752206
C	-4.374020	-4.815922	0.950384	C	-4.313314	-5.004304	0.658290
N	-3.281482	-4.216889	0.518386	N	-3.204615	-4.344077	0.388099
H	-5.390383	0.474108	0.255051	H	-5.458665	0.280815	0.172478
N	-1.664306	-1.745985	-0.466988	H	-4.408929	-6.073972	0.799469
H	-1.073411	-2.590222	-0.418256	N	-1.592549	-1.763405	-0.275343
H	-1.329716	-0.953284	-1.000507	H	-0.968238	-2.581569	-0.188813
H	-6.406013	-4.184068	1.344479	H	-1.229597	-0.918130	-0.696986
N	-0.555458	3.667823	-0.221977	N	-0.645953	3.614789	0.055018
C	-0.600329	5.002065	-0.384995	C	-0.685095	4.949039	-0.135447
N	-1.689096	5.775372	-0.554942	N	-1.765297	5.715396	-0.362914
C	-2.812710	5.044797	-0.555734	C	-2.886773	4.977376	-0.395357
C	-2.925323	3.652617	-0.401926	C	-3.006834	3.586913	-0.224966
C	-1.713874	2.950192	-0.216179	C	-1.798781	2.892492	0.028505
N	-4.125280	5.493532	-0.678599	N	-4.191141	5.419452	-0.575145
C	-4.938659	4.366818	-0.594502	C	-5.008772	4.299707	-0.502846
N	-4.262102	3.248554	-0.417496	N	-4.337992	3.183524	-0.294214
H	0.366134	5.504104	-0.375281	H	0.282400	5.448358	-0.099342
N	-1.648247	1.605961	-0.042701	H	-6.082167	4.396240	-0.612291
H	-0.785190	1.225641	0.325624	N	-1.735584	1.557225	0.241221
H	-2.490165	1.008525	-0.033526	H	-0.886743	1.189048	0.651759
H	-4.405008	6.442911	-0.894858	H	-2.563247	0.941119	0.182535
N	3.637519	0.549903	-0.088604	N	3.635179	0.656568	-0.012882
C	4.924940	0.509389	0.298980	C	4.958375	0.687353	0.244425
N	5.684381	1.531538	0.736232	N	5.717760	1.761055	0.521295
C	4.994825	2.680870	0.754585	C	4.984479	2.886231	0.527672
C	3.652989	2.877739	0.384123	C	3.605110	3.015452	0.286451
C	2.961548	1.732107	-0.066425	C	2.917623	1.813338	-0.012015
N	5.447191	3.944541	1.125369	N	5.423299	4.187209	0.740234
C	4.375267	4.816062	0.949185	C	4.312000	5.011210	0.616681
N	3.282348	4.217191	0.518004	N	3.204641	4.348230	0.346775
H	5.390507	-0.474034	0.253043	H	5.454696	-0.282068	0.223199
N	1.664098	1.746476	-0.466347	H	4.408084	6.083099	0.739209
H	1.073513	2.590919	-0.417725	N	1.596436	1.759670	-0.297230
H	1.329266	0.954074	-1.000186	H	0.968047	2.574934	-0.214550
H	6.407503	4.183972	1.341738	H	1.235481	0.903899	-0.698706
N	0.554865	-3.667300	-0.221840	N	0.649669	-3.619408	0.077371

C	0.599372	-5.001647	-0.384112	C	0.690100	-4.953639	-0.113406
N	1.687951	-5.775322	-0.553665	N	1.770167	-5.718147	-0.348102
C	2.811738	-5.045020	-0.554922	C	2.890251	-4.978412	-0.385908
C	2.924709	-3.652766	-0.401966	C	3.009379	-3.588276	-0.212442
C	1.713444	-2.949941	-0.216539	C	1.801303	-2.895321	0.045074
N	4.124188	-5.494166	-0.677461	N	4.194349	-5.418519	-0.573083
C	4.937862	-4.367619	-0.594024	C	5.010926	-4.297925	-0.500403
N	4.261597	-3.249066	-0.417818	N	4.339836	-3.183039	-0.286647
H	-0.367230	-5.503405	-0.374047	H	-0.276033	-5.454990	-0.071025
N	1.648056	-1.605555	-0.043909	H	6.083924	-4.392901	-0.614507
H	0.785316	-1.225054	0.324994	N	1.736454	-1.559222	0.254611
H	2.490151	-1.008372	-0.034563	H	0.889691	-1.193790	0.672616
H	4.403671	-6.443770	-0.893005	H	2.564700	-0.943881	0.198998
N	-4.516540	-6.181356	1.148951	N	-6.742760	-4.628777	1.020704
H	-3.624386	-6.666999	1.103741	H	-7.079851	-4.132710	1.850239
H	-5.064689	-6.457165	1.959180	H	-7.336016	-4.336956	0.239309
N	-6.313414	4.457739	-0.757808	N	-4.648367	6.746689	-0.762189
H	-6.778956	3.577067	-0.554114	H	-4.290830	7.304971	0.017877
H	-6.762772	5.244960	-0.297877	H	-4.202859	7.111722	-1.608905
N	4.518079	6.181467	1.147694	N	6.736207	4.636718	1.015777
H	3.625971	6.667179	1.103230	H	7.035924	4.213563	1.898827
H	5.066800	6.457158	1.957587	H	7.349570	4.264701	0.285809
N	6.312592	-4.459018	-0.757128	N	4.651903	-6.744489	-0.767218
H	6.778358	-3.578339	-0.553941	H	4.281983	-7.311246	0.000917
H	6.761676	-5.246083	-0.296674	H	4.220727	-7.100064	-1.624745

Cartesian coordinates of equilibrium geometries of C8-NO_2 and N9-NO_2 substituted derivatives of the A_4N3 tetramer

		C8-NO_2				N9-NO_2	
N	0.762105	-2.997601	-0.002039	N	0.799313	-2.963521	0.010903
C	-0.578206	-2.999225	-0.059629	C	-0.534085	-2.924275	-0.040614
N	-1.419908	-4.059815	-0.036391	N	-1.403494	-3.965737	-0.037838
C	-0.752158	-5.219820	0.061837	C	-0.762712	-5.136236	0.028657
C	0.654024	-5.368452	0.135661	C	0.624975	-5.332023	0.091592
C	1.430617	-4.173428	0.096003	C	1.437816	-4.164939	0.077500
N	-1.241600	-6.507720	0.119584	N	-1.276103	-6.451618	0.059465
C	-0.134223	-7.330055	0.224998	C	-0.176217	-7.333685	0.139607
N	1.015280	-6.693851	0.238115	N	0.957875	-6.688678	0.158914
N	2.773196	-4.171586	0.153559	N	2.780201	-4.184198	0.127636
H	-1.065189	-2.026954	-0.123311	H	-1.005436	-1.944114	-0.083269
H	3.263227	-5.053825	0.235659	H	-0.329387	-8.403161	0.177467
H	3.302994	-3.288643	0.115847	H	3.269847	-5.068731	0.176567
H	-2.204019	-6.829164	0.094854	H	3.315670	-3.303319	0.102994
N	-2.996881	-0.760308	-0.014188	N	-2.964151	-0.799955	-0.003116
C	-2.998740	0.580316	0.037428	C	-2.923994	0.533403	0.051222
N	-4.059900	1.421416	0.020093	N	-3.964902	1.403414	0.045906
C	-5.220345	0.752481	-0.064311	C	-5.135402	0.763214	-0.025804
C	-5.368833	-0.654287	-0.127679	C	-5.332002	-0.624311	-0.091174
C	-4.173205	-1.430091	-0.096832	C	-4.165303	-1.437767	-0.076350
N	-6.509017	1.240749	-0.114816	N	-6.450348	1.277435	-0.062302
C	-7.331591	0.132135	-0.204808	C	-7.332793	0.178390	-0.145793
N	-6.694834	-1.017016	-0.213638	N	-6.688477	-0.956146	-0.163219
N	-4.171222	-2.772999	-0.149312	N	-4.184074	-2.779847	-0.135836
H	-2.026336	1.068241	0.091559	H	-1.943722	1.004204	0.097293
H	-5.054737	-3.261589	-0.218608	H	-8.402035	0.332395	-0.187213

H	-3.287399	-3.301608	-0.118932	H	-5.069048	-3.267438	-0.191756
H	-6.830792	2.203143	-0.094996	H	-3.302823	-3.314325	-0.106735
N	-0.759776	2.997737	0.003785	N	-0.801308	2.964202	0.002640
C	0.580961	2.999483	-0.044991	C	0.531823	2.923806	-0.054912
N	1.422140	4.060497	-0.024282	N	1.402080	3.964435	-0.049171
C	0.753202	5.220823	0.061781	C	0.762773	5.134960	0.027703
C	-0.653653	5.369347	0.123871	C	-0.624380	5.331798	0.097495
C	-1.429593	4.173903	0.087656	C	-1.438315	4.165455	0.080041
N	1.241447	6.509371	0.114912	N	1.277713	6.449473	0.066958
C	0.132712	7.331824	0.205678	C	0.179380	7.332019	0.157047
N	-1.016528	6.695149	0.213498	N	-0.955389	6.688050	0.175580
N	-2.772600	4.172406	0.136196	N	-2.780392	4.184957	0.139416
H	1.068843	2.027051	-0.099322	H	1.001996	1.943430	-0.104446
H	-3.262605	5.055664	0.201473	H	0.333926	8.400993	0.201928
H	-3.302018	3.289220	0.098833	H	-3.268322	5.069596	0.194413
H	2.203901	6.831136	0.096307	H	-3.315573	3.304169	0.110295
N	2.998411	0.761256	0.009838	N	2.962692	0.799851	-0.010968
C	2.999771	-0.579197	0.064333	C	2.923196	-0.533440	0.044959
N	4.060160	-1.421043	0.038777	N	3.964572	-1.402950	0.043495
C	5.220249	-0.753228	-0.059034	C	5.135042	-0.762374	-0.026927
C	5.369129	0.653158	-0.129460	C	5.331056	0.625163	-0.092985
C	4.174284	1.429862	-0.087364	C	4.163979	1.438083	-0.080767
N	6.508043	-1.242721	-0.119529	N	6.450285	-1.275980	-0.060856
C	7.330578	-0.135184	-0.221938	C	7.332365	-0.176406	-0.144076
N	6.694585	1.014456	-0.231199	N	6.687527	0.957776	-0.163494
N	4.172626	2.772612	-0.141834	N	4.182829	2.780209	-0.137051
H	2.027394	-1.066136	0.126753	H	1.943073	-1.004633	0.090026
H	5.055067	3.261640	-0.224256	H	8.401742	-0.329876	-0.184184
H	3.289809	3.302394	-0.103494	H	5.067248	3.269211	-0.189890
H	6.829268	-2.205281	-0.098355	H	3.301509	3.314909	-0.114036
N	-0.313748	-8.772510	0.311842	N	-2.665018	-6.870839	0.013885
O	-1.503346	-9.169775	0.271397	O	-2.843737	-8.086337	0.071053
O	0.690182	-9.484238	0.416541	O	-3.498596	-5.977319	-0.076619
N	-8.775026	0.310163	-0.281266	N	-6.868902	2.666577	-0.020123
O	-9.172553	1.499879	-0.253283	O	-5.975133	3.499837	0.071509
O	-9.487292	-0.695415	-0.365394	O	-8.084245	2.845712	-0.081468
N	0.310522	8.775079	0.283860	N	2.666898	6.867612	0.019941
O	1.500329	9.172635	0.255462	O	3.499227	5.973743	-0.077366
O	-0.695260	9.487189	0.369966	O	2.846992	8.082611	0.083466
N	8.773027	-0.314698	-0.309565	N	6.869406	-2.664895	-0.016419
O	9.170058	-1.504488	-0.274638	O	5.975878	-3.498527	0.074912
O	9.484994	0.689537	-0.409269	O	8.084911	-2.843489	-0.075710

Cartesian coordinates of equilibrium geometries of C8-Cl and N9-Cl substituted derivatives of the A_4N3 tetramer

		C8-Cl				N9-Cl	
N	0.735872	-3.022739	0.001552	N	0.750554	-3.000770	0.025582
C	-0.604686	-3.017709	-0.040982	C	-0.588385	-2.985604	-0.048320
N	-1.444221	-4.079073	0.003686	N	-1.432707	-4.042876	-0.080110
C	-0.770387	-5.236444	0.107485	C	-0.765852	-5.203982	-0.023888
C	0.624595	-5.389078	0.163935	C	0.625017	-5.375259	0.060474
C	1.399902	-4.202396	0.103089	C	1.407151	-4.187659	0.080557
N	-1.268537	-6.530947	0.190454	N	-1.253768	-6.507534	-0.031862
C	-0.162179	-7.363293	0.292361	C	-0.163517	-7.376446	0.045644
N	0.982729	-6.733328	0.280105	N	0.974878	-6.723459	0.102667

N	2.749272	-4.200213	0.145023	N	2.753414	-4.193842	0.151967
H	-1.089943	-2.044803	-0.109207	H	-1.070373	-2.009577	-0.083291
H	3.236857	-5.082346	0.228342	H	-0.305554	-8.449429	0.055169
H	3.279620	-3.320679	0.093590	H	3.238433	-5.081158	0.183702
H	-2.240746	-6.815872	0.184804	H	3.288405	-3.314415	0.138125
N	-3.023003	-0.736871	0.004938	N	-3.000781	-0.749170	-0.054412
C	-3.017885	0.603669	0.049103	C	-2.985938	0.589890	0.016383
N	-4.079026	1.443303	0.002171	N	-4.043512	1.433266	0.062817
C	-5.236132	0.769550	-0.105967	C	-5.204584	0.764995	0.026242
C	-5.388805	-0.625426	-0.164601	C	-5.375677	-0.626169	-0.053033
C	-4.202350	-1.400849	-0.101105	C	-4.187680	-1.407147	-0.089621
N	-6.530364	1.267822	-0.191901	N	-6.508427	1.251526	0.055516
C	-7.362618	0.161568	-0.297819	C	-7.377189	0.160349	-0.005700
N	-6.732826	-0.983411	-0.285144	N	-6.724038	-0.977522	-0.072013
N	-4.199937	-2.750172	-0.145201	N	-4.193537	-2.753604	-0.158298
H	-2.045089	1.088727	0.121155	H	-2.010056	1.072941	0.036162
H	-5.081617	-3.237535	-0.229103	H	-8.450292	0.301147	0.002854
H	-3.320384	-3.280377	-0.090793	H	-5.080231	-3.239748	-0.173201
H	-6.815202	2.240068	-0.185645	H	-3.313518	-3.287921	-0.148095
N	-0.734625	3.022148	0.009933	N	-0.750604	3.001021	0.018315
C	0.606141	3.017981	-0.026371	C	0.588401	2.985947	-0.052749
N	1.444568	4.080436	0.013239	N	1.432953	4.043131	-0.080998
C	0.769187	5.237984	0.105126	C	0.766202	5.204210	-0.024574
C	-0.626261	5.389787	0.153621	C	-0.624770	5.375374	0.057239
C	-1.400278	4.202084	0.098135	C	-1.407093	4.187867	0.074218
N	1.265581	6.533633	0.180480	N	1.254324	6.507661	-0.029855
C	0.157895	7.365681	0.270801	C	0.163994	7.376516	0.046691
N	-0.986319	6.734537	0.257595	N	-0.974509	6.723524	0.100749
N	-2.749827	4.199375	0.131260	N	-2.753357	4.194113	0.143619
H	1.092494	2.044993	-0.085831	H	1.070260	2.009970	-0.087726
H	-3.237903	5.081563	0.208447	H	0.306170	8.449421	0.057887
H	-3.279400	3.318847	0.087959	H	-3.238048	5.081164	0.177987
H	2.237505	6.819578	0.177394	H	-3.288452	3.314711	0.130142
N	3.024148	0.735724	0.010038	N	3.000558	0.749679	-0.049984
C	3.019251	-0.604879	0.051882	C	2.985436	-0.589335	0.023553
N	4.080424	-1.444418	0.003567	N	4.042949	-1.432930	0.067082
C	5.237491	-0.770519	-0.103449	C	5.204108	-0.765098	0.024151
C	5.389954	0.624506	-0.160268	C	5.375463	0.625891	-0.058519
C	4.203466	1.399811	-0.095374	C	4.187540	1.407221	-0.091486
N	6.531811	-1.268639	-0.189286	N	6.507947	-1.252058	0.047943
C	7.363907	-0.162163	-0.293428	C	7.376896	-0.161336	-0.019435
N	6.734000	0.982749	-0.279855	N	6.723825	0.976700	-0.084463
N	4.201142	2.749275	-0.136996	N	4.193563	2.753583	-0.163226
H	2.046486	-1.090028	0.122572	H	2.009430	-1.072067	0.048042
H	5.082707	3.236817	-0.222991	H	8.450014	-0.302514	-0.015937
H	3.321840	3.279568	-0.082209	H	5.080653	3.239094	-0.183643
H	6.816849	-2.240830	-0.183460	H	3.313686	3.288034	-0.150667
Cl	-0.375914	-9.078668	0.425418	Cl	-2.917291	-6.962519	-0.142977
Cl	-9.077717	0.375499	-0.435187	Cl	-6.963283	2.914761	0.170198
Cl	0.369353	9.082260	0.392924	Cl	2.918087	6.962663	-0.137239
Cl	9.079134	-0.375840	-0.430009	Cl	6.962710	-2.915222	0.163645

Cartesian coordinates of equilibrium geometries of C8-F and N9-F substituted derivatives of the A_4N3 tetramer

C8-F				N9-F			
N	0.739801	-3.017661	0.023226	N	0.763584	-2.996309	0.002570
C	-0.599853	-3.009637	-0.011493	C	-0.576530	-2.980047	-0.046231
N	-1.438694	-4.073142	0.012762	N	-1.427185	-4.031583	-0.004670
C	-0.763794	-5.230032	0.083287	C	-0.756809	-5.188193	0.103031
C	0.630717	-5.385752	0.126962	C	0.637590	-5.365836	0.168493
C	1.404671	-4.200468	0.091961	C	1.420346	-4.179643	0.108859
N	-1.265313	-6.531714	0.131811	N	-1.213697	-6.488558	0.184631
C	-0.154187	-7.347460	0.200078	C	-0.152927	-7.368799	0.294530
N	0.990664	-6.740493	0.200299	N	0.988374	-6.708910	0.285133
N	2.755279	-4.197381	0.126455	N	2.766255	-4.184003	0.156925
H	-1.086299	-2.036316	-0.058725	H	-1.056271	-2.005053	-0.118455
H	3.243339	-5.081472	0.177200	H	-0.305901	-8.436860	0.372263
H	3.283023	-3.316058	0.083706	H	3.252828	-5.067478	0.234328
H	-2.235529	-6.823303	0.124568	H	3.298104	-3.304058	0.098595
N	-3.018162	-0.740259	-0.010856	N	-2.995109	-0.763189	-0.014299
C	-3.009933	0.599373	0.027992	C	-2.978835	0.576973	0.032262
N	-4.073045	1.438532	-0.000786	N	-4.030759	1.427394	-0.004255
C	-5.229751	0.764198	-0.081001	C	-5.187875	0.756605	-0.104501
C	-5.385599	-0.630141	-0.129743	C	-5.365617	-0.637943	-0.166291
C	-4.200751	-1.404525	-0.089080	C	-4.178959	-1.420351	-0.112148
N	-6.530926	1.266236	-0.138032	N	-6.488813	1.213007	-0.179818
C	-7.346494	0.155551	-0.215711	C	-7.369472	0.151816	-0.282164
N	-6.739839	-0.989519	-0.214703	N	-6.709311	-0.989301	-0.274711
N	-4.198055	-2.755075	-0.127867	N	-4.183277	-2.766273	-0.157549
H	-2.036636	1.085462	0.082124	H	-2.003654	1.057069	0.098500
H	-5.081245	-3.243130	-0.183723	H	-8.438022	0.304374	-0.353700
H	-3.316966	-3.282884	-0.080948	H	-5.067368	-3.252562	-0.230954
H	-6.822278	2.236540	-0.130273	H	-3.302787	-3.297696	-0.105067
N	-0.740405	3.018024	0.016186	N	-0.764132	2.995690	0.005576
C	0.599085	3.009878	-0.024271	C	0.576001	2.979297	-0.042590
N	1.438278	4.073071	0.002353	N	1.426641	4.030867	-0.001592
C	0.764156	5.229836	0.082086	C	0.756146	5.187576	0.105860
C	-0.630052	5.385626	0.132595	C	-0.638321	5.365271	0.171047
C	-1.404473	4.200715	0.094059	C	-1.421040	4.179089	0.110799
N	1.266289	6.531057	0.136166	N	1.212833	6.488023	0.186444
C	0.155710	7.346659	0.214466	C	0.151888	7.368409	0.296126
N	-0.989343	6.739991	0.216514	N	-0.989348	6.708495	0.287637
N	-2.754949	4.198048	0.133955	N	-2.766928	4.183642	0.156679
H	1.085012	2.036614	-0.078114	H	1.055776	2.004272	-0.114333
H	-3.243627	5.081290	0.190112	H	0.304730	8.436574	0.373018
H	-3.282981	3.317014	0.088943	H	-3.253758	5.067018	0.233184
H	2.236566	6.822442	0.126356	H	-3.299151	3.303866	0.097834
N	3.017216	0.740152	-0.023805	N	2.995683	0.763263	-0.016892
C	3.009062	-0.599374	0.013173	C	2.979473	-0.576971	0.029743
N	4.072416	-1.438452	-0.011124	N	4.031597	-1.427219	-0.005630
C	5.229369	-0.764052	-0.084091	C	5.188629	-0.756202	-0.105289
C	5.385218	0.630266	-0.130128	C	5.366259	0.638387	-0.167969
C	4.200153	1.404549	-0.094923	C	4.179588	1.420718	-0.113762
N	6.530830	-1.265998	-0.133375	N	6.489790	-1.212319	-0.178563
C	7.346685	-0.155227	-0.204520	C	7.370383	-0.151019	-0.281023
N	6.739958	0.989725	-0.206000	N	6.709981	0.989975	-0.275223

N	4.197476	2.755104	-0.130804	N	4.184269	2.766782	-0.156623
H	2.035717	-1.085516	0.062578	H	2.004216	-1.057257	0.095370
H	5.081242	3.243122	-0.183505	H	8.439041	-0.303405	-0.351519
H	3.316293	3.283178	-0.089199	H	5.067597	3.253943	-0.232891
H	6.822199	-2.236258	-0.125286	H	3.303664	3.298376	-0.103272
F	-0.334902	-8.674415	0.260215	F	-2.560773	-6.862075	0.168551
F	-8.672847	0.336831	-0.285824	F	-6.862439	2.560066	-0.165257
F	0.337078	8.673092	0.281927	F	2.559943	6.861481	0.170409
F	8.673424	-0.336463	-0.266202	F	6.863856	-2.559209	-0.161154

Cartesian coordinates of equilibrium geometries of C8-H / N9-H substituted derivatives of the A_4N3 tetramer

		C8-H / N9-H	
N	0.736640	-3.015814	0.007581
C	-0.605347	-3.011075	-0.027876
N	-1.443541	-4.071621	0.011378
C	-0.769091	-5.232895	0.100710
C	0.626882	-5.386283	0.148535
C	1.399524	-4.196133	0.094868
N	-1.265048	-6.524378	0.171611
C	-0.167218	-7.372604	0.258535
N	0.981700	-6.728928	0.246885
N	2.749669	-4.194339	0.129786
H	-1.090573	-2.037506	-0.086329
H	-0.286596	-8.447247	0.327640
H	3.234053	-5.079048	0.202607
H	3.280720	-3.314275	0.082310
N	-3.016778	-0.737370	0.006564
C	-3.012008	0.604562	0.044687
N	-4.071994	1.443060	-0.002410
C	-5.232580	0.768955	-0.103467
C	-5.385806	-0.626900	-0.155171
C	-4.196417	-1.399912	-0.092440
N	-6.523309	1.265306	-0.185121
C	-7.371055	0.167706	-0.280847
N	-6.727776	-0.981344	-0.265766
N	-4.194611	-2.750067	-0.129595
H	-2.038794	1.089435	0.112293
H	-8.445018	0.287424	-0.358721
H	-5.078529	-3.233660	-0.213214
H	-3.314778	-3.281229	-0.079444
N	-0.736728	3.016131	0.010790
C	0.605261	3.011474	-0.026603
N	1.443385	4.072138	0.011726
C	0.768900	5.233386	0.102074
C	-0.627031	5.386661	0.152017
C	-1.399641	4.196442	0.099159
N	1.264762	6.524978	0.172278
C	0.166990	7.373172	0.260550
N	-0.981848	6.729326	0.250676
N	-2.749781	4.194499	0.135871
H	1.090505	2.037889	-0.086310
H	0.286346	8.447865	0.329259
H	-3.234262	5.078788	0.209967
H	-3.280793	3.314494	0.087313

N	3.016311	0.736870	0.001849
C	3.011319	-0.605137	0.038303
N	4.071439	-1.443575	-0.005104
C	5.232492	-0.769383	-0.100513
C	5.386105	0.626547	-0.149725
C	4.196389	1.399479	-0.091032
N	6.523496	-1.265647	-0.177696
C	7.371734	-0.167980	-0.268906
N	6.728484	0.981133	-0.254912
N	4.194959	2.749614	-0.126594
H	2.037898	-1.090078	0.101601
H	8.445996	-0.287607	-0.343129
H	5.079852	3.233289	-0.199854
H	3.315321	3.281052	-0.076882
H	-2.243081	-6.788354	0.165498
H	-6.787082	2.243397	-0.180026
H	2.242740	6.789070	0.164662
H	6.787239	-2.243751	-0.171897

Cartesian coordinates of equilibrium geometries of C8-Me and N9-Me substituted derivatives of the A_4N3 tetramer

	C8-Me				N9-Me		
N	0.732237	-3.021613	-0.022270	N	0.755783	-3.006819	-0.000775
C	-0.608990	-3.015581	-0.058305	C	-0.585663	-2.998129	-0.052526
N	-1.446970	-4.078168	-0.032672	N	-1.425401	-4.057645	-0.028369
C	-0.772202	-5.239504	0.039093	C	-0.752688	-5.220685	0.062833
C	0.622727	-5.393774	0.083772	C	0.642646	-5.377828	0.127562
C	1.394265	-4.204591	0.049791	C	1.416487	-4.188486	0.089599
N	-1.266826	-6.532808	0.092292	N	-1.268580	-6.505177	0.118594
C	-0.171509	-7.395189	0.167683	C	-0.171191	-7.354237	0.213815
N	0.974719	-6.739358	0.163192	N	0.986601	-6.721846	0.221067
N	2.745880	-4.201695	0.090177	N	2.767106	-4.183831	0.143522
H	-1.094788	-2.041599	-0.104275	H	-1.069028	-2.023903	-0.110610
H	3.230215	-5.086401	0.157632	H	-0.301935	-8.428930	0.274131
H	3.276076	-3.320683	0.067603	H	3.252496	-5.068013	0.214872
H	-2.245674	-6.793505	0.082782	H	3.294000	-3.300243	0.102420
N	-3.019974	-0.732317	0.012827	N	-3.005350	-0.754919	0.001755
C	-3.013800	0.608991	0.045653	C	-2.996188	0.586772	0.047389
N	-4.076403	1.446968	0.021265	N	-4.055776	1.426463	0.023419
C	-5.238042	0.772086	-0.045303	C	-5.219304	0.753463	-0.060267
C	-5.392599	-0.623036	-0.086061	C	-5.377050	-0.642276	-0.116422
C	-4.203272	-1.394489	-0.053852	C	-4.187579	-1.416006	-0.079737
N	-6.531366	1.266731	-0.095106	N	-6.503725	1.269312	-0.115802
C	-7.394209	0.171299	-0.164960	C	-7.353447	0.171564	-0.201830
N	-6.738568	-0.975020	-0.160448	N	-6.721427	-0.986453	-0.204900
N	-4.200223	-2.746157	-0.090572	N	-4.183767	-2.766918	-0.125748
H	-2.039671	1.094824	0.088222	H	-2.021728	1.070396	0.100724
H	-5.085469	-3.230737	-0.154806	H	-8.428246	0.302280	-0.259778
H	-3.319148	-3.276376	-0.069291	H	-5.068643	-3.252045	-0.190362
H	-6.791848	2.245660	-0.086598	H	-3.300051	-3.294135	-0.093743
N	-0.732565	3.021460	-0.020126	N	-0.755398	3.006740	-0.005826
C	0.608616	3.015658	-0.057328	C	0.586124	2.997855	-0.054508
N	1.446431	4.078345	-0.031358	N	1.425970	4.057277	-0.028449
C	0.771539	5.239528	0.041603	C	0.753191	5.220472	0.061465
C	-0.623384	5.393567	0.087250	C	-0.642364	5.377880	0.122029

C	-1.394776	4.204283	0.053458	C	-1.416234	4.188647	0.082364
N	1.266011	6.532892	0.094553	N	1.269235	6.504699	0.120126
C	0.170607	7.395125	0.169892	C	0.171693	7.354021	0.212825
N	-0.975490	6.739116	0.166587	N	-0.986262	6.721973	0.216166
N	-2.746309	4.200816	0.096340	N	-2.767023	4.184216	0.131734
H	1.094432	2.041721	-0.104560	H	1.069508	2.023585	-0.111837
H	-3.231065	5.085320	0.165050	H	0.302585	8.428603	0.274448
H	-3.275810	3.319385	0.069100	H	-3.251776	5.068736	0.205306
H	2.244787	6.793748	0.084602	H	-3.293518	3.300176	0.097104
N	3.020398	0.731513	0.013899	N	3.005232	0.755981	-0.000473
C	3.014749	-0.609715	0.050451	C	2.996024	-0.585539	0.048500
N	4.077713	-1.447301	0.028384	N	4.055311	-1.425594	0.023217
C	5.239031	-0.772095	-0.040555	C	5.218663	-0.753038	-0.066661
C	5.392959	0.622884	-0.085196	C	5.376355	0.642397	-0.128426
C	4.203412	1.393996	-0.054675	C	4.187324	1.416568	-0.088549
N	6.532664	-1.266236	-0.090324	N	6.502945	-1.269243	-0.123425
C	7.394833	-0.170589	-0.163757	C	7.352526	-0.171921	-0.216323
N	6.738629	0.975405	-0.161438	N	6.720622	0.986094	-0.221712
N	4.200092	2.745636	-0.095416	N	4.183511	2.767381	-0.136596
H	2.040785	-1.095836	0.093785	H	2.021658	-1.068780	0.105664
H	5.085534	3.229447	-0.160683	H	8.427197	-0.302974	-0.276500
H	3.318881	3.275471	-0.074549	H	5.067324	3.252380	-0.213723
H	6.793693	-2.245009	-0.079967	H	3.299804	3.294518	-0.100844
C	-0.334431	-8.880640	0.246125	C	-2.685368	-6.858440	0.084460
H	-0.897642	-9.174080	1.142510	H	-3.204749	-6.428771	0.948137
H	-0.870598	-9.271363	-0.629403	H	-3.146304	-6.474132	-0.831953
H	0.654535	-9.343841	0.286975	H	-2.782040	-7.947012	0.108591
C	-8.879845	0.334534	-0.238355	C	-6.856147	2.686573	-0.090600
H	-9.266793	0.875286	0.636057	H	-6.484282	3.150801	0.829390
H	-9.176453	0.893516	-1.136402	H	-6.413833	3.202134	-0.950171
H	-9.343801	-0.654409	-0.272493	H	-7.944181	2.784126	-0.130546
C	0.333477	8.880688	0.246090	C	2.686452	6.857183	0.094391
H	0.897030	9.175666	1.141728	H	3.201797	6.419372	0.956437
H	0.869280	9.269933	-0.630329	H	3.150967	6.480597	-0.823495
H	-0.655516	9.343820	0.286498	H	2.783851	7.945441	0.128608
C	8.880569	-0.332924	-0.238221	C	6.855348	-2.686373	-0.093948
H	9.269617	-0.865678	0.640133	H	6.477024	-3.148851	0.824241
H	9.176358	-0.899162	-1.131881	H	6.419231	-3.203676	-0.955650
H	9.343244	0.656158	-0.281517	H	7.943653	-2.783808	-0.125821

Cartesian coordinates of equilibrium geometries of C8-NH_2 and N9-NH_2 substituted derivatives of the A_4N3 tetramer

		C8-NH_2				N9-NH_2	
N	0.713832	-3.025822	0.124576	N	0.781520	-2.992879	-0.423647
C	-0.625599	-3.015279	0.090101	C	-0.560517	-2.987877	-0.419921
N	-1.461049	-4.080932	0.022071	N	-1.394812	-4.041796	-0.258847
C	-0.784341	-5.239080	-0.002709	C	-0.711661	-5.187804	-0.095307
C	0.608360	-5.398061	0.032150	C	0.682528	-5.344126	-0.076640
C	1.376509	-4.211565	0.094531	C	1.449191	-4.162125	-0.247408
N	-1.286967	-6.537786	-0.079437	N	-1.209473	-6.471261	0.092330
C	-0.185198	-7.389531	-0.078094	C	-0.111177	-7.315905	0.211029
N	0.965440	-6.749434	-0.025216	N	1.037775	-6.677120	0.117547
N	2.730938	-4.209896	0.126162	N	2.800710	-4.155773	-0.246256
H	-1.114002	-2.042077	0.118197	H	-1.050856	-2.024779	-0.555508
H	3.217582	-5.095396	0.099190	H	-0.250876	-8.379172	0.363267

H	3.260220	-3.328662	0.134098	H	3.285215	-5.028915	-0.086740
H	-2.262315	-6.800546	-0.006126	H	3.323890	-3.270859	-0.296830
N	-3.025267	-0.723413	0.103739	N	-3.031021	-0.772975	-0.466190
C	-3.007654	0.615526	0.148773	C	-3.036565	0.567949	-0.514891
N	-4.067503	1.459197	0.098135	N	-4.084941	1.401927	-0.320944
C	-5.226812	0.792106	-0.004028	C	-5.212675	0.719338	-0.058289
C	-5.392797	-0.599073	-0.059250	C	-5.355373	-0.673818	0.027944
C	-4.211855	-1.376439	-0.002500	C	-4.180999	-1.439656	-0.188447
N	-6.519578	1.306794	-0.092615	N	-6.485584	1.216611	0.193871
C	-7.375738	0.212889	-0.191038	C	-7.310596	0.118958	0.414296
N	-6.742938	-0.943025	-0.185332	N	-6.669622	-1.028934	0.325829
N	-4.215741	-2.730497	-0.053724	N	-4.162316	-2.790590	-0.135409
H	-2.033167	1.096049	0.225121	H	-2.084368	1.057057	-0.715823
H	-5.101476	-3.209832	-0.130807	H	-8.362546	0.258200	0.631899
H	-3.338703	-3.265355	-0.023010	H	-5.006925	-3.270429	0.145406
H	-6.779402	2.279288	0.019317	H	-3.271621	-3.303204	-0.199066
N	-0.712792	3.026204	0.124109	N	-0.783207	2.998178	-0.466459
C	0.626837	3.016325	0.095469	C	0.558781	2.991970	-0.470568
N	1.462081	4.082208	0.027569	N	1.394790	4.040751	-0.287247
C	0.784921	5.240028	-0.002902	C	0.713759	5.182591	-0.090367
C	-0.607992	5.398305	0.025927	C	-0.680011	5.339513	-0.059663
C	-1.375903	4.211585	0.087749	C	-1.448768	4.162970	-0.255475
N	1.287105	6.538842	-0.080724	N	1.214105	6.460006	0.129238
C	0.184818	7.389929	-0.086092	C	0.117129	7.301972	0.277661
N	-0.965621	6.749283	-0.036557	N	-1.032936	6.667251	0.172764
N	-2.730461	4.209697	0.113060	N	-2.800456	4.157672	-0.245293
H	1.115574	2.043401	0.128478	H	1.047678	2.031809	-0.629701
H	-3.217340	5.095151	0.076959	H	0.258687	8.360444	0.458838
H	-3.259788	3.328455	0.120319	H	-3.282080	5.023738	-0.043927
H	2.261897	6.802501	-0.002633	H	-3.323943	3.272888	-0.298265
N	3.026353	0.723514	0.119755	N	3.028697	0.774694	-0.482278
C	3.008886	-0.615427	0.166235	C	3.034954	-0.566467	-0.522219
N	4.068314	-1.459256	0.110393	N	4.083782	-1.398995	-0.324590
C	5.227122	-0.792449	0.000992	C	5.211220	-0.714237	-0.066198
C	5.392879	0.598638	-0.056508	C	5.353247	0.679469	0.011622
C	4.212386	1.376220	0.006077	C	4.178680	1.443598	-0.209697
N	6.519191	-1.307390	-0.095055	N	6.482807	-1.209517	0.196061
C	7.374878	-0.213616	-0.200045	C	7.306151	-0.110242	0.415261
N	6.742239	0.942340	-0.191595	N	6.665846	1.036981	0.313709
N	4.216484	2.730241	-0.046421	N	4.159418	2.794566	-0.163567
H	2.034688	-1.095750	0.247455	H	2.082487	-1.057201	-0.716566
H	5.102097	3.209392	-0.129351	H	8.356477	-0.247895	0.641754
H	3.339851	3.265576	-0.013919	H	5.006663	3.277384	0.104724
H	6.779699	-2.279693	0.017224	H	3.268563	3.307029	-0.227233
N	-0.345634	-8.770839	-0.060074	N	-2.558989	-6.889890	0.150529
H	-1.049590	-9.136227	-0.696802	H	-3.008251	-6.402440	0.929861
H	0.544427	-9.250731	-0.167612	H	-3.021496	-6.583435	-0.709250
N	-8.755609	0.381653	-0.214991	N	-6.912044	2.564579	0.224020
H	-9.093331	1.130413	-0.815172	H	-6.369306	3.053010	0.941079
H	-9.236491	-0.495636	-0.398124	H	-6.678541	2.988652	-0.677432
N	0.344430	8.771487	-0.070264	N	2.564233	6.874414	0.197726
H	1.048027	9.135923	-0.707794	H	3.014673	6.361408	0.960226
H	-0.546043	9.249914	-0.180362	H	3.024176	6.597084	-0.672983
N	8.754520	-0.382644	-0.233116	N	6.908357	-2.557403	0.242269
H	9.088139	-1.131583	-0.834948	H	6.332327	-3.046061	0.932851
H	9.233973	0.493867	-0.420221	H	6.716037	-2.980884	-0.669705

Cartesian coordinates of equilibrium geometries of C2-NO_2 and N9-NO_2 substituted derivatives of the A_4N7 tetramer

C2-NO_2				N9-NO_2			
N	-2.882640	-4.070288	0.112133	N	-3.068526	-4.272155	-0.041273
C	-2.941541	-5.388763	0.234678	C	-3.117654	-5.616488	-0.043280
N	-1.987965	-6.313372	0.262797	N	-2.099527	-6.502509	-0.010282
C	-0.779413	-5.734954	0.160238	C	-0.917402	-5.885231	0.025904
C	-0.532251	-4.357882	0.035753	C	-0.702488	-4.500784	0.033213
C	-1.658911	-3.500939	-0.006785	C	-1.851892	-3.672570	0.000013
N	0.480399	-6.316385	0.166491	N	0.396124	-6.411100	0.069403
C	1.406070	-5.293324	0.060873	C	1.290475	-5.336719	0.097697
N	0.825715	-4.106441	-0.016880	N	0.657454	-4.188920	0.075236
N	-1.573983	-2.160288	-0.146981	N	-1.770637	-2.324778	0.008828
H	2.479965	-5.453698	0.045689	H	-4.115923	-6.051269	-0.075084
H	-0.705109	-1.761276	-0.475273	H	2.363162	-5.475276	0.128921
H	-2.461917	-1.623706	-0.173139	H	-0.856762	-1.897639	0.054932
H	0.677690	-7.308568	0.243331	H	-2.617938	-1.737320	-0.018678
N	-4.069601	2.882747	-0.108369	N	-4.269261	3.068450	0.047739
C	-5.387709	2.942153	-0.234254	C	-5.613508	3.119722	0.044813
N	-6.312608	1.988953	-0.264933	N	-6.500908	2.102995	0.007368
C	-5.734892	0.780157	-0.161235	C	-5.885315	0.919983	-0.027796
C	-4.358258	0.532423	-0.033226	C	-4.501164	0.702842	-0.030523
C	-3.500980	1.658763	0.011861	C	-3.671370	1.851003	0.007257
N	-6.316771	-0.479431	-0.169790	N	-6.413184	-0.392578	-0.075341
C	-5.294333	-1.405535	-0.061938	C	-5.340118	-1.288676	-0.099235
N	-4.107454	-0.825677	0.019281	N	-4.191380	-0.657575	-0.073196
N	-2.160841	1.573475	0.155746	N	-2.323612	1.767940	0.004498
H	-5.455179	-2.479369	-0.047812	H	-6.046811	4.118648	0.076166
H	-1.762233	0.703953	0.482735	H	-5.480474	-2.361055	-0.131018
H	-1.623625	2.461028	0.180669	H	-1.897131	0.853915	-0.045368
H	-7.308830	-0.676290	-0.249381	H	-1.735072	2.614982	0.030079
N	2.882582	4.070264	0.109697	N	3.068087	4.268739	-0.052181
C	2.941529	5.388695	0.232607	C	3.118498	5.613055	-0.052276
N	1.987943	6.313257	0.261952	N	2.101464	6.500017	-0.012387
C	0.779309	5.734802	0.160488	C	0.918942	5.883824	0.029230
C	0.532088	4.357761	0.035879	C	0.702748	4.499607	0.035463
C	1.658752	3.500887	-0.008201	C	1.851121	3.670275	-0.005292
N	-0.480528	6.316163	0.168234	N	-0.393852	6.410902	0.080291
C	-1.406263	5.293088	0.063272	C	-1.289090	5.337270	0.110540
N	-0.825922	4.106256	-0.015358	N	-0.657276	4.188899	0.083090
N	1.573835	2.160366	-0.149094	N	1.768542	2.322541	0.000440
H	-2.480180	5.453419	0.049114	H	4.116977	6.046914	-0.088712
H	0.704345	1.761120	-0.475457	H	-2.361482	5.476894	0.146578
H	2.461653	1.623622	-0.175192	H	0.854619	1.896510	0.056718
H	-0.677785	7.308323	0.245476	H	2.614903	1.734327	-0.028815
N	4.069890	-2.882717	-0.110295	N	4.268873	-3.067405	0.043529
C	5.388126	-2.942008	-0.235057	C	5.613160	-3.118274	0.042965
N	6.312924	-1.988693	-0.265361	N	6.500388	-2.101387	0.005798
C	5.734977	-0.779935	-0.162484	C	5.884552	-0.918581	-0.032125
C	4.358212	-0.532347	-0.035553	C	4.500373	-0.701914	-0.037540
C	3.501038	-1.658775	0.009078	C	3.670746	-1.850149	0.000751
N	6.316694	0.479729	-0.170809	N	6.412035	0.394127	-0.080474
C	5.294064	1.405727	-0.063885	C	5.338702	1.289798	-0.107758
N	4.107200	0.825725	0.016556	N	4.190191	0.658268	-0.082678

N	2.160721	-1.573539	0.151646	N	2.323012	-1.767192	-0.003286
H	5.454755	2.479586	-0.049804	H	6.046686	-4.117028	0.076303
H	1.762043	-0.704193	0.479022	H	5.478529	2.362204	-0.140986
H	1.623703	-2.461203	0.177429	H	1.896691	-0.853234	-0.055640
H	7.308779	0.676710	-0.249667	H	1.734973	-2.614136	0.022406
N	-4.384873	-5.936439	0.350813	N	0.818810	-7.828320	0.082593
O	-4.517008	-7.148960	0.527328	O	-0.078075	-8.647655	0.019355
O	-5.308662	-5.113330	0.255693	O	2.032764	-7.992630	0.155864
N	-5.934524	4.385752	-0.351818	N	-7.831094	-0.813111	-0.087362
O	-7.146921	4.518469	-0.528524	O	-8.648960	0.085079	-0.024366
O	-5.110880	5.309160	-0.257591	O	-7.997415	-2.026906	-0.159984
N	4.384961	5.936405	0.347542	N	-0.815161	7.828450	0.096081
O	4.517276	7.149135	0.522557	O	0.082381	8.647015	0.032103
O	5.308654	5.113103	0.253035	O	-2.028822	7.993862	0.172174
N	5.935242	-4.385581	-0.351356	N	7.829741	0.815184	-0.090968
O	7.148022	-4.518264	-0.525634	O	8.647896	-0.082643	-0.026354
O	5.111436	-5.309008	-0.258547	O	7.995665	2.028963	-0.164083

Cartesian coordinates of equilibrium geometries of C2-Cl and N9-Cl substituted derivatives of the A_4N7 tetramer

	C2-Cl				N9-Cl		
N	-3.016211	-4.262713	-0.213764	N	-3.041396	-4.286097	-0.191212
C	-3.040985	-5.598041	-0.251981	C	-3.078520	-5.630134	-0.297912
N	-2.044970	-6.482725	-0.186497	N	-2.054160	-6.504557	-0.311429
C	-0.860270	-5.855976	-0.073505	C	-0.878480	-5.876807	-0.208931
C	-0.654410	-4.471218	-0.024698	C	-0.672399	-4.495495	-0.095125
C	-1.809660	-3.652036	-0.093139	C	-1.833424	-3.679593	-0.081477
N	0.409578	-6.405688	0.015533	N	0.416262	-6.401952	-0.193761
C	1.304727	-5.353350	0.108144	C	1.311126	-5.346056	-0.080645
N	0.699543	-4.179471	0.086589	N	0.682808	-4.188803	-0.020133
N	-1.750948	-2.305165	-0.048012	N	-1.763518	-2.334077	0.029633
H	2.377107	-5.491283	0.183776	H	-4.073381	-6.066566	-0.380868
H	-0.856787	-1.876221	0.145288	H	2.384049	-5.489452	-0.050813
H	-2.612411	-1.730493	-0.065843	H	-0.865191	-1.915561	0.226205
H	0.629841	-7.395355	0.009641	H	-2.617353	-1.754806	0.046238
N	-4.264338	3.015289	0.198164	N	-4.283354	3.041609	0.188651
C	-5.599483	3.037191	0.245745	C	-5.626606	3.078809	0.304493
N	-6.482443	2.039657	0.180212	N	-6.501272	2.054693	0.320147
C	-5.854038	0.856865	0.056239	C	-5.874589	0.879183	0.210046
C	-4.469171	0.654217	-0.003273	C	-4.494087	0.672990	0.086862
C	-3.652037	1.810839	0.065497	C	-3.678004	1.833873	0.070680
N	-6.401737	-0.413672	-0.035970	N	-6.400249	-0.415324	0.195223
C	-5.348237	-1.306185	-0.138688	C	-5.345373	-1.310262	0.073644
N	-4.175526	-0.698522	-0.121999	N	-4.188317	-0.682161	0.007160
N	-2.305239	1.755471	0.009874	N	-2.333393	1.764301	-0.050757
H	-5.484327	-2.378643	-0.216567	H	-6.062067	4.073500	0.394094
H	-1.876213	0.865507	-0.201445	H	-5.489293	-2.383097	0.043109
H	-1.732799	2.618104	0.034553	H	-1.916126	0.866939	-0.253924
H	-7.390871	-0.636070	-0.024758	H	-1.754257	2.618347	-0.063288
N	3.015365	4.259621	-0.188982	N	3.041195	4.283471	-0.184091
C	3.040863	5.594701	-0.236340	C	3.079400	5.626905	-0.297934
N	2.045439	6.480282	-0.174740	N	2.055772	6.502119	-0.315389
C	0.860543	5.854922	-0.056591	C	0.879606	5.875828	-0.209802
C	0.653925	4.470564	0.000811	C	0.672394	4.495214	-0.089352
C	1.808762	3.650408	-0.061919	C	1.832788	3.678526	-0.070745

N	-0.408905	6.405905	0.029691	N	-0.414707	6.402049	-0.197982
C	-1.304684	5.354619	0.127776	C	-1.310466	5.347356	-0.080607
N	-0.700181	4.180311	0.113132	N	-0.683094	4.189901	-0.013979
N	1.749738	2.303775	-0.005114	N	1.761991	2.333752	0.048485
H	-2.377049	5.493655	0.201258	H	4.074583	6.062042	-0.383885
H	0.857637	1.876927	0.202781	H	-2.383297	5.491749	-0.052737
H	2.611330	1.729211	-0.023254	H	0.863839	1.917029	0.249430
H	-0.628592	7.395649	0.018007	H	2.615670	1.754248	0.064352
N	4.263759	-3.015743	0.205288	N	4.283744	-3.041538	0.185607
C	5.598706	-3.037933	0.258570	C	5.627111	-3.078777	0.300326
N	6.481982	-2.040004	0.203523	N	6.501477	-2.054472	0.319944
C	5.854112	-0.856358	0.085193	C	5.874316	-0.878660	0.215573
C	4.469573	-0.653242	0.022232	C	4.493652	-0.672408	0.094398
C	3.652100	-1.810311	0.078912	C	3.677926	-1.833475	0.073415
N	6.402158	0.414798	0.003444	N	6.399476	0.416099	0.205956
C	5.349057	1.308028	-0.097440	C	5.344199	1.311171	0.088939
N	4.176277	0.700324	-0.088415	N	4.187303	0.682922	0.020675
N	2.305647	-1.754091	0.017326	N	2.333383	-1.763660	-0.047643
H	5.485396	2.380958	-0.169028	H	6.062981	-4.073712	0.385141
H	1.877719	-0.861086	-0.184025	H	5.487719	2.384157	0.062469
H	1.732035	-2.616138	0.031312	H	1.915519	-0.864727	-0.242742
H	7.391259	0.637069	0.019016	H	1.753851	-2.617345	-0.062373
Cl	-4.665444	-6.302292	-0.409510	Cl	0.836254	-8.070185	-0.318894
Cl	-6.306122	4.659294	0.416423	Cl	-8.067953	-0.835025	0.328504
Cl	4.665351	6.296941	-0.400746	Cl	-0.833283	8.070160	-0.330113
Cl	6.304562	-4.661074	0.422294	Cl	8.067213	0.835840	0.339146

Cartesian coordinates of equilibrium geometries of C2-F and N9-F substituted derivatives of the A_4N7 tetramer

		C2-F				N9-F	
N	-3.031888	-4.260080	-0.068338	N	-3.052185	-4.293960	-0.061429
C	-3.046596	-5.590828	-0.043781	C	-3.092615	-5.642605	-0.053767
N	-2.063643	-6.479536	0.036587	N	-2.073827	-6.520399	0.012596
C	-0.873953	-5.851746	0.096648	C	-0.898614	-5.883642	0.070107
C	-0.666855	-4.466465	0.082202	C	-0.683300	-4.495748	0.068869
C	-1.822241	-3.647625	-0.001982	C	-1.844558	-3.681639	0.004282
N	0.395161	-6.399291	0.182899	B	0.396639	-6.373199	0.141887
C	1.294457	-5.345342	0.212467	C	1.306170	-5.341506	0.175320
N	0.690218	-4.173344	0.154126	N	0.670923	-4.182171	0.132939
N	-1.759730	-2.301401	-0.021777	N	-1.772108	-2.333409	0.002480
H	2.367761	-5.483487	0.270249	H	-4.088340	-6.081425	-0.107232
H	-0.851991	-1.867344	0.063340	H	2.377174	-5.492168	0.222921
H	-2.617273	-1.721255	-0.071496	H	-0.863938	-1.902539	0.098499
H	0.613747	-7.388778	0.212216	H	-2.622027	-1.749550	-0.041445
N	-4.262566	3.032086	0.063181	N	-4.294787	3.052567	0.060142
C	-5.593391	3.045288	0.041975	C	-5.643488	3.092026	0.054127
N	-6.481304	2.061116	-0.034252	N	-6.520747	2.072555	-0.011427
C	-5.852375	0.871941	-0.093754	C	-5.883229	0.897754	-0.070090
C	-4.466811	0.666411	-0.082868	C	-4.495189	0.683452	-0.070713
C	-3.649004	1.822907	-0.002777	C	-3.681773	1.845256	-0.006821
N	-6.398745	-0.398059	-0.175940	N	-6.371922	-0.397991	-0.141824
C	-5.343881	-1.296251	-0.206754	C	-5.339509	-1.306682	-0.176417
N	-4.172395	-0.690533	-0.153358	N	-4.180545	-0.670530	-0.135156
N	-2.302687	1.761770	0.012915	N	-2.333547	1.773323	-0.007246
H	-5.480809	-2.369884	-0.262083	H	-6.082938	4.087501	0.108273

H	-1.867813	0.854164	-0.070146	H	-5.489258	-2.377837	-0.224174
H	-1.722575	2.619182	0.062476	H	-1.902428	0.865190	-0.103905
H	-7.388061	-0.617863	-0.202116	H	-1.749205	2.622314	0.036685
N	3.032333	4.262422	-0.063592	N	3.052371	4.294902	-0.059064
C	3.046130	5.593219	-0.041328	C	3.091910	5.643600	-0.052990
N	2.062352	6.481467	0.034698	N	2.072387	6.520897	0.011560
C	0.872875	5.853011	0.092206	C	0.897448	5.883475	0.068993
C	0.666628	4.467549	0.079523	C	0.683067	4.495433	0.069578
C	1.822877	3.649312	0.000340	C	1.844945	3.681934	0.007205
N	-0.396807	6.399905	0.173553	N	-0.398373	6.372283	0.138970
C	-1.295586	5.345480	0.202613	C	-1.307248	5.339993	0.172566
N	-0.690514	4.173769	0.148779	N	-0.671085	4.181006	0.132453
N	1.761287	2.303014	-0.016477	N	1.773015	2.333691	0.008939
H	-2.369131	5.483028	0.257036	H	4.087485	6.082997	-0.106006
H	0.853536	1.868457	0.064935	H	-2.378501	5.489837	0.218829
H	2.619395	1.723304	-0.065434	H	0.864886	1.902472	0.106107
H	-0.616009	7.389289	0.200593	H	2.621350	1.748519	-0.034667
N	4.262060	-3.032977	0.067540	N	4.295806	-3.052476	0.059673
C	5.592834	-3.046840	0.043552	C	5.644495	-3.091796	0.053175
N	6.481019	-2.063184	-0.035098	N	6.521565	-2.072090	-0.011159
C	5.852539	-0.873775	-0.093706	C	5.883881	-0.897279	-0.067840
C	4.467102	-0.667487	-0.079749	C	4.495796	-0.683104	-0.068017
C	3.648927	-1.823604	0.002349	C	3.682577	-1.845177	-0.005937
N	6.399401	0.395800	-0.177586	N	6.372460	0.398628	-0.137255
C	5.344971	1.294550	-0.206587	C	5.340032	1.307344	-0.170134
N	4.173269	0.689573	-0.150365	N	4.181130	0.671030	-0.130197
N	2.302626	-1.761981	0.021109	N	2.334308	-1.773633	-0.007304
H	5.482533	2.368018	-0.262639	H	6.084091	-4.087304	0.105613
H	1.868039	-0.854308	-0.062002	H	5.489731	2.378620	-0.215801
H	1.723433	-2.619144	0.071406	H	1.902736	-0.865635	-0.103663
H	7.388777	0.614957	-0.205995	H	1.749212	-2.622763	0.036053
F	-4.293056	-6.129693	-0.113067	F	0.744366	-7.719414	0.166620
F	-6.133448	4.291351	0.110140	F	-7.717937	-0.746829	-0.165415
F	4.292414	6.132815	-0.108038	F	-0.747164	7.718305	0.161787
F	6.132439	-4.293051	0.111430	F	7.718433	0.747599	-0.160152

Cartesian coordinates of equilibrium geometries of C2-H / N9-H substituted derivatives of the A_4N7 tetramer

		C2-H / N9-H	
N	-3.055222	-4.331704	-0.054295
C	-3.077421	-5.680540	-0.037879
N	-2.046794	-6.544174	0.027301
C	-0.871341	-5.896006	0.076876
C	-0.685539	-4.506732	0.067874
C	-1.853122	-3.703843	0.001665
N	0.412514	-6.422090	0.145532
C	1.290986	-5.354193	0.171074
N	0.668023	-4.190352	0.126495
N	-1.796914	-2.354129	-0.010741
H	-4.068782	-6.131237	-0.082814
H	2.367498	-5.471244	0.217717
H	-0.890410	-1.915867	0.069859
H	-2.648942	-1.770743	-0.047899
N	-4.329216	3.054890	0.055997
C	-5.677995	3.079275	0.036890

N	-6.543088	2.050102	-0.029825
C	-5.896719	0.873583	-0.077070
C	-4.507777	0.685450	-0.064430
C	-3.703136	1.851816	0.002115
N	-6.424781	-0.409294	-0.145958
C	-5.358310	-1.289610	-0.168458
N	-4.193567	-0.668680	-0.121610
N	-2.353483	1.793700	0.016924
H	-6.127175	4.071341	0.080721
H	-5.477248	-2.365911	-0.214455
H	-1.916519	0.886657	-0.064039
H	-1.769794	2.645639	0.052994
N	3.056059	4.331517	-0.058644
C	3.079037	5.680312	-0.040456
N	2.048848	6.544445	0.026245
C	0.872992	5.896865	0.074997
C	0.686338	4.507702	0.063768
C	1.853585	3.704262	-0.003337
N	-0.410604	6.423660	0.144665
C	-1.289757	5.356271	0.168442
N	-0.667530	4.192066	0.121963
N	1.796792	2.354589	-0.016712
H	4.070685	6.130495	-0.085122
H	-2.366237	5.474002	0.215331
H	0.890096	1.916628	0.062778
H	2.649170	1.770827	-0.053761
N	4.327441	-3.054986	0.062845
C	5.676156	-3.080075	0.040066
N	6.541496	-2.051559	-0.032020
C	5.895557	-0.874894	-0.080906
C	4.506749	-0.686056	-0.064722
C	3.701776	-1.851822	0.007654
N	6.424007	0.407419	-0.155413
C	5.357900	1.288189	-0.178103
N	4.193038	0.667981	-0.125678
N	2.352168	-1.793126	0.027172
H	6.124972	-4.072208	0.085663
H	5.477283	2.364256	-0.228073
H	1.915323	-0.886259	-0.055449
H	1.769158	-2.645782	0.063707
H	0.647594	-7.407802	0.166450
H	-7.410826	-0.642628	-0.169025
H	-0.645086	7.409493	0.167197
H	7.410095	0.640113	-0.182083

Cartesian coordinates of equilibrium geometries of C2-Me and N9-Me substituted derivatives of the A_4N7 tetramer

	C2-Me				N9-Me		
N	-3.046491	-4.333552	0.001361	N	-3.041476	-4.326437	-0.126051
C	-3.090428	-5.688314	0.030697	C	-3.060363	-5.675069	-0.157773
N	-2.042411	-6.540536	0.059975	N	-2.027930	-6.538395	-0.118198
C	-0.866212	-5.893614	0.058741	C	-0.852997	-5.888851	-0.041802
C	-0.675559	-4.507331	0.031550	C	-0.671466	-4.499596	-0.003105
C	-1.846583	-3.707622	0.000105	C	-1.840303	-3.697704	-0.045134
N	0.417687	-6.424252	0.088083	N	0.427504	-6.428467	0.009808

C	1.301232	-5.358914	0.078914	C	1.298464	-5.355830	0.076548
N	0.679935	-4.194118	0.045013	N	0.679435	-4.186362	0.069720
N	-1.790826	-2.357512	-0.031258	N	-1.787483	-2.347981	-0.011242
H	2.378285	-5.478848	0.096656	H	-4.050858	-6.125728	-0.222025
H	-0.880567	-1.920283	-0.045012	H	2.375025	-5.477263	0.122576
H	-2.644268	-1.775071	-0.044525	H	-0.884161	-1.912917	0.112103
H	0.649344	-7.410566	0.109989	H	-2.642216	-1.766341	-0.027608
N	-4.334091	3.045785	-0.004351	N	-4.326038	3.041071	0.123907
C	-5.688898	3.089295	-0.032018	C	-5.674665	3.059748	0.156605
N	-6.540779	2.040924	-0.058187	N	-6.537889	2.027180	0.117257
C	-5.893383	0.864976	-0.056357	C	-5.888238	0.852318	0.039641
C	-4.506922	0.674782	-0.031888	C	-4.498968	0.671036	-0.000613
C	-3.707528	1.846199	-0.002525	C	-3.697196	1.839954	0.041793
N	-6.423634	-0.419156	-0.082181	N	-6.427658	-0.428313	-0.012104
C	-5.357983	-1.302357	-0.072688	C	-5.354919	-1.299063	-0.080386
N	-4.193334	-0.680654	-0.044846	N	-4.185595	-0.679770	-0.074384
N	-2.357167	1.791423	0.027085	N	-2.347422	1.787216	0.007518
H	-5.477536	-2.379475	-0.086566	H	-6.125418	4.050220	0.221511
H	-1.919733	0.881245	0.043473	H	-5.476087	-2.375656	-0.126955
H	-1.775163	2.644732	0.040113	H	-1.912305	0.884132	-0.118413
H	-7.409938	-0.651194	-0.101217	H	-1.765558	2.641253	0.023797
N	3.046693	4.333484	0.000246	N	3.041562	4.328409	-0.117079
C	3.090912	5.688250	0.030329	C	3.059361	5.677017	-0.150987
N	2.043051	6.540641	0.060438	N	2.026134	6.539572	-0.114768
C	0.866737	5.893922	0.059146	C	0.851552	5.889234	-0.039237
C	0.675791	4.507674	0.031047	C	0.671155	4.499899	0.001802
C	1.846673	3.707741	-0.000770	C	1.840726	3.698861	-0.037123
N	-0.417060	6.424819	0.089296	N	-0.429576	6.427837	0.008770
C	-1.300824	5.359663	0.079203	C	-1.299733	5.354571	0.075853
N	-0.679794	4.194720	0.044149	N	-0.679630	4.185658	0.072390
N	1.790677	2.357619	-0.031675	N	1.788791	2.349128	-0.001269
H	-2.377850	5.479845	0.097049	H	4.049627	6.128385	-0.214107
H	0.880350	1.920603	-0.045576	H	-2.376524	5.475069	0.119752
H	2.643608	1.774653	-0.045547	H	0.885574	1.913554	0.121416
H	-0.648520	7.411176	0.112148	H	2.643347	1.767638	-0.017667
N	4.333299	-3.045801	-0.002800	N	4.328237	-3.042072	0.118534
C	5.688076	-3.089692	-0.031283	C	5.676854	-3.060265	0.151510
N	6.540242	-2.041596	-0.059181	N	6.539647	-2.027233	0.115411
C	5.893221	-0.865451	-0.058012	C	5.889520	-0.852420	0.041433
C	4.506860	-0.674843	-0.032236	C	4.500182	-0.671563	0.001574
C	3.707188	-1.845972	-0.001410	C	3.698909	-1.841010	0.039721
N	6.423851	0.418498	-0.086171	N	6.428466	0.428576	-0.005730
C	5.358467	1.301999	-0.077336	C	5.355386	1.299114	-0.070913
N	4.193650	0.680663	-0.045773	N	4.186259	0.679411	-0.067314
N	2.356974	-1.790472	0.029434	N	2.349172	-1.788808	0.004095
H	5.478294	2.379076	-0.093825	H	6.128018	-4.050705	0.213613
H	1.919769	-0.880218	0.044643	H	5.476230	2.375906	-0.113690
H	1.775222	-2.644066	0.042760	H	1.913674	-0.885346	-0.117358
H	7.410199	0.650228	-0.106817	H	1.767301	-2.643032	0.020269
C	-4.472680	-6.302594	0.025991	C	0.753722	-7.854771	0.010156
H	-5.055538	-5.929116	0.877112	H	0.397004	-8.325032	0.932724
H	-5.009817	-6.014284	-0.886631	H	0.270357	-8.343090	-0.841441
H	-4.407443	-7.391948	0.078536	H	1.837463	-7.971010	-0.065649
C	-6.303660	4.471364	-0.028872	C	-7.853871	-0.754937	-0.011250
H	-6.014918	5.009895	0.882781	H	-8.341482	-0.272501	0.841259
H	-5.931014	5.053182	-0.881056	H	-8.325199	-0.397585	-0.932981

H	-7.393031	4.405679	-0.080481	H	-7.969653	-1.838733	0.063748
C	4.473242	6.302382	0.025634	C	-0.757402	7.853796	0.005195
H	5.056594	5.927933	0.875986	H	-0.404018	8.326475	0.927760
H	5.009798	6.015031	-0.887623	H	-0.272149	8.340874	-0.846014
H	4.408187	7.391677	0.079364	H	-1.840997	7.968559	-0.074073
C	6.302454	-4.471907	-0.027175	C	7.854543	0.755910	-0.003112
H	6.013229	-5.009976	0.884608	H	8.342233	0.269687	0.847202
H	5.929923	-5.053948	-0.879266	H	8.326249	0.403191	-0.926440
H	7.391869	-4.406525	-0.078522	H	7.969759	1.839398	0.077027

Cartesian coordinates of equilibrium geometries of C2-NH_2 and N9-NH_2 substituted derivatives of the A_4N7 tetramer

	C2-NH_2				N9-NH_2		
N	-3.071790	-4.332025	0.021371	N	-2.989439	-4.321744	-0.290951
C	-3.102319	-5.688209	0.082708	C	-3.015410	-5.662563	-0.157248
N	-2.058948	-6.551183	0.125502	N	-1.983134	-6.517576	-0.011182
C	-0.887935	-5.901111	0.092127	C	-0.812301	-5.862361	-0.018153
C	-0.698575	-4.514580	0.028569	C	-0.619055	-4.482981	-0.156372
C	-1.871116	-3.714317	-0.007927	C	-1.786914	-3.689596	-0.291914
N	0.395929	-6.429121	0.117591	N	0.473729	-6.386458	0.112413
C	1.281433	-5.360671	0.069263	C	1.355987	-5.323195	0.047665
N	0.657729	-4.200100	0.016323	N	0.735735	-4.167108	-0.114534
N	-1.809081	-2.365642	-0.069801	N	-1.736424	-2.345060	-0.431471
H	2.357921	-5.480805	0.077563	H	-4.007026	-6.114651	-0.165506
H	-0.895120	-1.936551	-0.096289	H	2.428077	-5.456040	0.126956
H	-2.655769	-1.775823	-0.115878	H	-0.842362	-1.893608	-0.295700
H	0.628421	-7.414329	0.156405	H	-2.600256	-1.776782	-0.412941
N	-4.322272	3.057898	0.135173	N	-4.312189	2.950842	0.251981
C	-5.679524	3.086638	0.156716	C	-5.624853	2.933267	0.554110
N	-6.543042	2.046058	0.078785	N	-6.471065	1.883877	0.587108
C	-5.892498	0.880855	-0.037828	C	-5.835836	0.745063	0.273419
C	-4.504527	0.694183	-0.074756	C	-4.484498	0.598152	-0.060294
C	-3.703728	1.863459	0.016646	C	-3.701589	1.780662	-0.070758
N	-6.421077	-0.399275	-0.138800	N	-6.353611	-0.548799	0.221470
C	-5.351575	-1.280303	-0.227149	C	-5.312159	-1.391861	-0.122457
N	-4.190138	-0.657115	-0.191041	N	-4.177009	-0.738313	-0.299885
N	-2.353258	1.804686	-0.003344	N	-2.387375	1.776722	-0.384088
H	-5.471793	-2.354024	-0.305625	H	-6.060680	3.900516	0.803821
H	-1.924177	0.901329	-0.145863	H	-5.441710	-2.463536	-0.212831
H	-1.765953	2.654747	0.010490	H	-1.973306	0.910855	-0.698856
H	-7.406963	-0.631972	-0.147483	H	-1.815676	2.636428	-0.326753
N	3.070527	4.334261	0.040455	N	2.979654	4.327292	-0.149054
C	3.099998	5.690637	0.098523	C	3.004729	5.666434	0.001080
N	2.055946	6.552875	0.139456	N	1.970401	6.521999	0.128751
C	0.885437	5.901817	0.107227	C	0.798977	5.869931	0.078323
C	0.697152	4.514945	0.046659	C	0.606571	4.493035	-0.086805
C	1.870441	3.715469	0.012759	C	1.776362	3.698142	-0.193444
N	-0.398774	6.429043	0.129734	N	-0.488734	6.395719	0.175988
C	-1.283612	5.359950	0.082878	C	-1.371757	5.336565	0.066724
N	-0.659040	4.199695	0.033965	N	-0.749860	4.181128	-0.092531
N	1.809856	2.366517	-0.045127	N	1.728084	2.354950	-0.346613
H	-2.360167	5.479615	0.088608	H	3.997059	6.116226	0.026739
H	0.896484	1.935822	-0.064302	H	-2.445338	5.471893	0.115896
H	2.657095	1.777250	-0.090524	H	0.830668	1.903519	-0.234406
H	-0.631860	7.414232	0.165484	H	2.591827	1.787414	-0.302533

N	4.323858	-3.060041	0.125769	N	4.331638	-2.964695	0.164070
C	5.681008	-3.088626	0.154029	C	5.656729	-2.960119	0.406291
N	6.544560	-2.047136	0.089651	N	6.502715	-1.911510	0.461053
C	5.894280	-0.881152	-0.019827	C	5.853071	-0.758462	0.240763
C	4.506465	-0.694462	-0.061718	C	4.487597	-0.596691	-0.020488
C	3.705624	-1.864809	0.014777	C	3.706594	-1.779737	-0.064150
N	6.422959	0.399771	-0.107415	N	6.366281	0.538017	0.244696
C	5.353743	1.281384	-0.193493	C	5.310190	1.396361	-0.001588
N	4.192287	0.657692	-0.168322	N	4.169130	0.749716	-0.170629
N	2.355343	-1.806301	-0.013067	N	2.380896	-1.760381	-0.320483
H	5.474387	2.355674	-0.262835	H	6.103796	-3.938528	0.581394
H	1.926381	-0.900976	-0.142663	H	5.434612	2.471912	-0.037422
H	1.767518	-2.655842	-0.002647	H	1.946863	-0.869931	-0.520360
H	7.408844	0.632615	-0.109424	H	1.806739	-2.619480	-0.282979
N	-4.361302	-6.257633	0.065385	N	0.853049	-7.738317	0.290162
H	-4.419988	-7.224897	0.356833	H	0.377133	-8.084391	1.128147
H	-5.132139	-5.651861	0.314284	H	0.479656	-8.271344	-0.500400
N	-6.248202	4.344570	0.232805	N	-7.680789	-0.970940	0.474335
H	-7.223522	4.377712	0.502913	H	-7.915411	-0.694638	1.431925
H	-5.650770	5.086101	0.576075	H	-8.296867	-0.437167	-0.144758
N	4.358606	6.260921	0.079280	N	-0.869069	7.745919	0.368351
H	4.417377	7.228598	0.370757	H	-0.465912	8.055861	1.257442
H	5.130398	5.655779	0.327024	H	-0.416326	8.300277	-0.363731
N	6.249365	-4.346888	0.222726	N	7.703866	0.945908	0.469168
H	7.223176	-4.382969	0.497236	H	8.020697	0.485650	1.327223
H	5.650002	-5.092052	0.553858	H	8.274830	0.568313	-0.291958

Cartesian coordinates of equilibrium geometries substituted C2- NO_2, C8- NO_2 and N9- NO_2 derivatives of adenine 9H

	C2-NO_2				C8-NO_2		
N	-2.978850	-4.278003	-0.303890	N	-3.638244	-0.662093	-0.229155
C	-3.012150	-5.582720	-0.053149	C	-4.981924	-0.677291	-0.113788
N	-2.046080	-6.459376	0.190784	N	-5.807003	-1.733964	0.015828
C	-0.852392	-5.840674	0.180708	C	-5.125999	-2.885688	0.019032
C	-0.619902	-4.475890	-0.071809	C	-3.722264	-3.035261	-0.096424
C	-1.764340	-3.681463	-0.334055	C	-2.971190	-1.834834	-0.222135
N	0.401176	-6.374950	0.404416	N	-5.602626	-4.176056	0.135427
C	1.307614	-5.330845	0.278402	C	-4.492121	-4.995930	0.085137
N	0.733604	-4.178405	-0.006591	N	-3.348922	-4.360996	-0.052636
N	-1.690338	-2.360480	-0.615791	H	-5.461550	0.300477	-0.127092
H	2.371805	-5.486757	0.407353	N	-1.622542	-1.832485	-0.324692
H	-0.795463	-1.890224	-0.613272	H	-1.108143	-2.703172	-0.354090
H	-2.542021	-1.831794	-0.753080	H	-1.136466	-0.951431	-0.432484
H	0.603677	-7.345430	0.616842	H	-6.562304	-4.490754	0.238429
N	-4.413269	-6.174489	-0.035170	N	-4.654563	-6.439930	0.186394
O	-4.611993	-7.155501	-0.761734	O	-5.835767	-6.842335	0.312078
O	-5.236894	-5.627373	0.707382	O	-3.642645	-7.148466	0.143675

	N9-NO_2		
N	-3.591864	-0.639604	-0.269938
C	-4.933295	-0.615886	-0.191457
N	-5.789117	-1.651291	-0.062028
C	-5.140021	-2.815370	-0.011988
C	-3.750718	-3.004836	-0.078250

C	-2.963867	-1.836913	-0.214336
N	-5.631487	-4.134115	0.118932
C	-4.524000	-5.000539	0.122334
N	-3.394701	-4.352999	0.005653
H	-5.391469	0.370989	-0.239747
H	-4.661403	-6.068927	0.213298
N	-1.611248	-1.876037	-0.288439
H	-1.122103	-2.760680	-0.269504
H	-1.097393	-1.012268	-0.405730
N	-7.023315	-4.584044	0.225862
O	-7.157209	-5.800789	0.349525
O	-7.874011	-3.712431	0.179318

Cartesian coordinates of equilibrium geometries substituted C2-Cl, C8-Cl and N9-Cl derivatives of adenine 9H

	C2-Cl				C8-Cl		
N	-2.982693	-4.254082	-0.228673	N	-3.639915	-0.660538	-0.212706
C	-3.023574	-5.588934	-0.179258	C	-4.983229	-0.667517	-0.110784
N	-2.031168	-6.471324	-0.060169	N	-5.809212	-1.723182	0.020379
C	-0.843697	-5.845578	0.013071	C	-5.125628	-2.874909	0.043205
C	-0.620751	-4.458967	-0.024000	C	-3.732107	-3.030138	-0.052152
C	-1.775147	-3.650121	-0.150687	C	-2.979185	-1.840429	-0.184581
N	0.416821	-6.399609	0.137062	N	-5.613431	-4.170388	0.163420
C	1.317488	-5.341128	0.168391	C	-4.504930	-5.002754	0.135319
N	0.734036	-4.163359	0.073932	N	-3.364127	-4.374818	0.007821
N	-1.717278	-2.296571	-0.193474	H	-5.460626	0.311293	-0.138893
H	2.383777	-5.508405	0.260405	N	-1.623556	-1.838875	-0.273908
H	-0.822958	-1.826024	-0.164499	H	-1.114087	-2.710817	-0.316253
H	-2.569767	-1.765932	-0.317156	H	-1.141752	-0.961872	-0.422754
H	0.626419	-7.389709	0.195762	H	-6.583173	-4.449768	0.258633
Cl	-4.649220	-6.289333	-0.293363	Cl	-4.704862	-6.719284	0.266758

	N9-Cl		
N	-3.631700	-0.662832	-0.307890
C	-4.975645	-0.658028	-0.203392
N	-5.805889	-1.701711	-0.021796
C	-5.129913	-2.852518	0.053493
C	-3.738129	-3.025140	-0.033713
C	-2.980329	-1.843670	-0.224496
N	-5.604521	-4.149990	0.230501
C	-4.507001	-5.009805	0.241447
N	-3.372661	-4.363580	0.084191
H	-5.448702	0.320426	-0.277159
H	-4.638253	-6.076922	0.366450
N	-1.625653	-1.858682	-0.316847
H	-1.124399	-2.736987	-0.313332
H	-1.134797	-0.994063	-0.503158
Cl	-7.258538	-4.609199	0.409204

Cartesian coordinates of equilibrium geometries substituted C2-F, C8-F and N9-F derivatives of adenine 9H

	C2-F				C8-F		
N	-2.985738	-4.256464	-0.240968	N	-3.642757	-0.669902	-0.301539
C	-3.011337	-5.586743	-0.191960	C	-4.983548	-0.670780	-0.185020
N	-2.031519	-6.473836	-0.071644	N	-5.806979	-1.721261	0.002865

C	-0.842903	-5.847194	0.007849	C	-5.122537	-2.868143	0.068705
C	-0.623717	-4.459718	-0.025840	C	-3.730408	-3.028486	-0.033032
C	-1.779373	-3.651372	-0.155955	C	-2.980807	-1.848078	-0.225489
N	0.416325	-6.398365	0.139429	N	-5.612088	-4.162737	0.247417
C	1.316302	-5.337030	0.178701	C	-4.499433	-4.976561	0.239966
N	0.730944	-4.161777	0.082018	N	-3.360339	-4.376807	0.078971
N	-1.723281	-2.297999	-0.193368	H	-5.462825	0.304953	-0.252886
H	2.382133	-5.503446	0.278015	N	-1.624201	-1.846978	-0.322226
H	-0.829130	-1.827476	-0.162818	H	-1.115657	-2.719783	-0.352433
H	-2.574359	-1.768387	-0.323819	H	-1.150562	-0.979571	-0.537705
H	0.626804	-7.387948	0.199340	H	-6.578598	-4.445468	0.361650
F	-4.255434	-6.122053	-0.281637	F	-4.667213	-6.296539	0.394257

		N9-F	
N	-3.626711	-0.652616	-0.212447
C	-4.971635	-0.648150	-0.113202
N	-5.809209	-1.693218	0.016671
C	-5.131832	-2.846820	0.039669
C	-3.737559	-3.025928	-0.051957
C	-2.977635	-1.836863	-0.182889
N	-5.574935	-4.150980	0.157419
C	-4.507567	-5.026889	0.132497
N	-3.371509	-4.367863	0.006691
H	-5.440288	0.334683	-0.142464
H	-4.650053	-6.096757	0.209438
N	-1.623995	-1.852714	-0.273339
H	-1.123638	-2.731282	-0.283627
H	-1.126249	-0.980178	-0.393714
F	-6.907895	-4.538767	0.274759

Cartesian coordinates of equilibrium geometries substituted C2-H / C8-H / N9-H derivatives of adenine 9H

		C2-H / C8-H / N9-H	
N	-3.634160	-0.656588	-0.193865
C	-4.979778	-0.662677	-0.101923
N	-5.807593	-1.715775	0.017707
C	-5.126372	-2.872367	0.039636
C	-3.731862	-3.029213	-0.045946
C	-2.978063	-1.837521	-0.165020
N	-5.614481	-4.163923	0.147690
C	-4.515988	-5.012037	0.123071
N	-3.369796	-4.371588	0.007447
H	-5.454780	0.317409	-0.129367
H	-4.629204	-6.087167	0.193006
N	-1.620387	-1.840907	-0.235793
H	-1.121422	-2.717388	-0.303310
H	-1.136060	-0.967993	-0.401667
H	-6.590718	-4.422600	0.231836

Cartesian coordinates of equilibrium geometries substituted C2-Me, C8-Me and N9-Me derivatives of adenine 9H

		C2-Me				C8-Me	
N	-2.978969	-4.265416	-0.176116	N	-3.630197	-0.657202	-0.264202
C	-3.059990	-5.617088	-0.092603	C	-4.974141	-0.662384	-0.161619
N	-2.031892	-6.482829	0.033041	N	-5.800124	-1.716521	-0.024440
C	-0.844877	-5.857926	0.069586	C	-5.118086	-2.871457	0.002997

C	-0.616364	-4.474725	-0.005862	C	-3.724908	-3.028456	-0.091577
C	-1.772507	-3.667926	-0.129754	C	-2.974196	-1.839218	-0.227579
N	0.419339	-6.412430	0.186231	N	-5.603473	-4.162921	0.126063
C	1.323533	-5.357665	0.174910	C	-4.507517	-5.023918	0.101660
N	0.742586	-4.180740	0.061167	N	-3.364981	-4.371579	-0.028861
N	-1.705657	-2.308914	-0.185513	H	-5.450798	0.316730	-0.195734
H	2.390874	-5.525856	0.253050	N	-1.613610	-1.838907	-0.301948
H	-0.805477	-1.855373	-0.265620	H	-1.118795	-2.714706	-0.401737
H	-2.549574	-1.790160	-0.391389	H	-1.140227	-0.970136	-0.512052
H	0.626546	-7.401005	0.265690	H	-6.579560	-4.418607	0.220428
C	-4.454084	-6.199657	-0.135977	C	-4.662692	-6.507713	0.214019
H	-5.013072	-5.897566	0.759432	H	-5.153831	-6.785880	1.155849
H	-5.001793	-5.812075	-1.002573	H	-5.267798	-6.908126	-0.610227
H	-4.411831	-7.290534	-0.180355	H	-3.674524	-6.972521	0.182464

N9-Me

N	-3.626489	-0.665754	-0.275160
C	-4.974571	-0.676151	-0.235032
N	-5.801746	-1.730623	-0.118255
C	-5.115767	-2.883592	-0.038531
C	-3.717163	-3.034388	-0.063443
C	-2.966300	-1.841785	-0.186474
N	-5.617290	-4.169818	0.083875
C	-4.510686	-5.007679	0.128008
N	-3.356708	-4.371755	0.041093
H	-5.451952	0.300633	-0.308043
H	-4.628711	-6.081238	0.222067
N	-1.605783	-1.835118	-0.198927
H	-1.099659	-2.709236	-0.242164
H	-1.124412	-0.965376	-0.385825
C	-7.030555	-4.532050	0.173684
H	-7.475174	-4.100090	1.076286
H	-7.569914	-4.150148	-0.698703
H	-7.116644	-5.621028	0.209047

Cartesian coordinates of equilibrium geometries substituted C2-NH_2, C8-NH_2 and N9-NH_2 derivatives of adenine 9H

C2-NH_2				C8-NH_2			
N	-2.985327	-4.244441	-0.121774	N	-3.628862	-0.669289	-0.273969
C	-3.058527	-5.598244	-0.021824	C	-4.972567	-0.663341	-0.217595
N	-2.038442	-6.478702	0.103858	N	-5.805565	-1.714433	-0.068472
C	-0.851829	-5.857080	0.108032	C	-5.129904	-2.865272	0.017972
C	-0.620075	-4.474370	0.009643	C	-3.735688	-3.032159	-0.027100
C	-1.773692	-3.662696	-0.104413	C	-2.979218	-1.852196	-0.173566
N	0.412050	-6.413115	0.206679	N	-5.633367	-4.155001	0.172501
C	1.323541	-5.358888	0.160764	C	-4.534296	-5.008672	0.199494
N	0.742005	-4.184073	0.043650	N	-3.380508	-4.376716	0.095831
N	-1.692751	-2.303240	-0.173981	H	-5.446059	0.313777	-0.304009
H	2.391331	-5.531743	0.218597	N	-1.610099	-1.853622	-0.181904
H	-0.786311	-1.872127	-0.298525	H	-1.128841	-2.734166	-0.312718
H	-2.527067	-1.779592	-0.406218	H	-1.146348	-1.003983	-0.480115
H	0.618706	-7.401499	0.290213	H	-6.612551	-4.412823	0.134695
N	-4.327379	-6.131495	-0.092455	N	-4.692779	-6.384309	0.259802
H	-4.439645	-7.091431	0.206892	H	-3.808325	-6.865143	0.402004
H	-5.100731	-5.495972	0.051017	H	-5.420904	-6.721390	0.883461

N9-NH_2

N	-2.908042	-4.251952	0.426168
C	-2.885502	-5.570280	0.704248
N	-1.856412	-6.432286	0.592853
C	-0.755298	-5.813027	0.142061
C	-0.618901	-4.456802	-0.192953
C	-1.779003	-3.663426	-0.030639
N	0.501529	-6.357435	-0.098843
C	1.306640	-5.321857	-0.554924
N	0.673422	-4.167067	-0.623934
N	-1.801906	-2.337577	-0.329376
H	-3.824182	-5.989884	1.064376
H	2.343440	-5.505257	-0.810767
H	-0.949318	-1.865544	-0.595478
H	-2.634088	-1.801443	-0.122152
N	0.927152	-7.696269	0.068121
H	0.763652	-7.956283	1.045011
H	0.318951	-8.289400	-0.503616
